# Finite-time complete periodic synchronization of memristive neural networks with mixed delays

**DOI:** 10.1038/s41598-023-37737-2

**Published:** 2023-08-02

**Authors:** Hajer Brahmi, Boudour Ammar, Amel Ksibi, Farouk Cherif, Ghadah Aldehim, Adel M. Alimi

**Affiliations:** 1 Research Groups on Intelligent Machines, National Engineering School of Sfax, University of Sfax, 3038 Sfax, Tunisia; 2 Department of Information Systems, College of Computer and Information Sciences, Princess Nourah bint Abdulrahman University, P.O. Box 84428, Riyadh, 11671 Saudi Arabia; 3 Laboratory of Math Physics, Specials Functions and Applications LR11ES35, Department of Mathematics, ESSTHS, University of Sousse, Tunisia; 4 Department of Electrical and Electronic Engineering Science, Faculty of Engineering and the Built Environment, University of Johannesburg, South Africa

**Keywords:** Applied mathematics, Dynamical systems

## Abstract

In this paper we study the oscillatory behavior of a new class of memristor based neural networks with mixed delays and we prove the existence and uniqueness of the periodic solution of the system based on the concept of Filippov solutions of the differential equation with discontinuous right-hand side. In addition, some assumptions are determined to guarantee the globally exponentially stability of the solution. Then, we study the adaptive finite-time complete periodic synchronization problem and by applying Lyapunov–Krasovskii functional approach, a new adaptive controller and adaptive update rule have been developed. A useful finite-time complete synchronization condition is established in terms of linear matrix inequalities. Finally, an illustrative simulation is given to substantiate the main results.

## Introduction

Recurrent neural network (RNN) is a deep learning model characterized by a topology that allows all connections between neurons, and by the fact each neuron usually has a complicated structure because of a large number of parallel connections with a diversity of axon lengths^1,2^. In addition, RNNs are well known for their capacity of classification, detecting regularities and their ability to learn. They can play the role of memory through feedback and are perfectly able to receive sensory data from our future agent^3,4^. In particular, continuous time RNNs (CTRNNs) are RNNs modeled by dynamical systems in the form of differential equation; they combine machine learning and physical modeling^5–7^. In fact, CTRNNs are mathematically easier to analyze, and continuous formulation offers more flexibility in adapting the system to the problem and adding constraints. Actually, researchers are attracted to the mathematical properties of RNNs, namely, the nature of solutions, stability and the oscillation properties^8,9^.

Indeed, the dynamic properties of RNNs have been deeply discussed and several important works have been provided^10–13^. In particular, RNNs which exhibit periodic oscillation have been used to encode information in the vibration phase and to model many systems in many domains such as celestial mechanics, nonlinear vibration, electromagnetic theory, engineering, robotics^14–19^. In addition, the synchronization problem consists of analyzing the behavior between two systems: driver (or master) and responder (or slave) and could be seen in different real systems such as secure communication, information science, chaos generators and signal generators design, image processing, biological systems^20,21^. In fact, neuronal synchronization becomes one of the most attractive subjects in neuroscience, it indicates that the specific states of all the neurons in the neural networks converge to a common value^22–25^.

**Figure 1:**
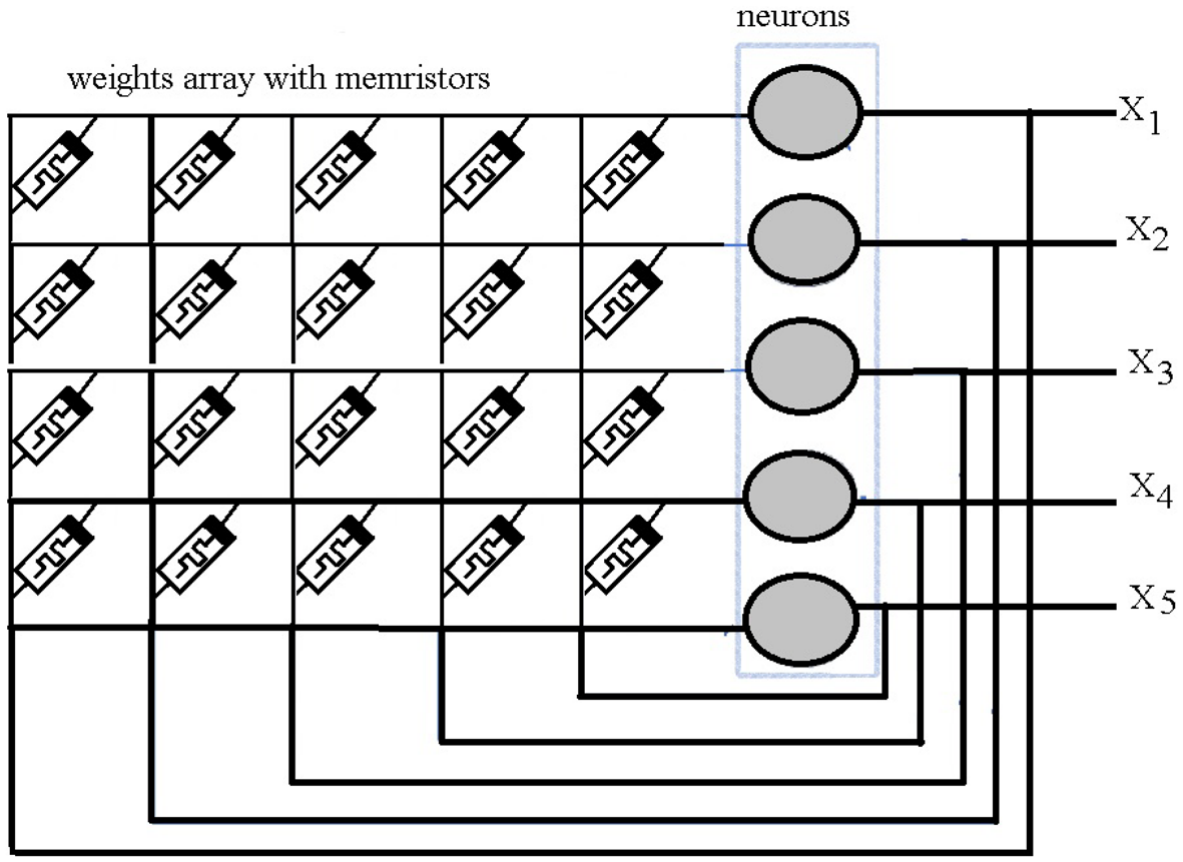
Memristive neural network with 5 neurons.

To make these oscillating neurons, researchers are interested in the memristor component that is a combination of memory and resistor^26,27^. Chua pointed out that the behaviour of memristor is somewhat similar to the synapses in the human brain^28^, and it can potentially offer both high connectivity and high density needed for efficient computing compared to other storage. A memristive neural networks (MNN) is described in Fig. 1. In addition, memristor studies show that MNN exhibits the feature of pinched hysteresis which means that a lag occurs between the application and the removal of a field and its subsequent effect, just as the neurons in the human brain have^29–31^. Some studies have been discussed to analyze the dynamic behaviour of MNN and a lot of researches were released^32–35^.

Hence, one can ask what is the impact of the delays (time-varying and distributed delay) for the stability and the synchronization of the periodic solution of MNNs. In Ref.^10^, authors investigate whether periodic solutions exist, are unique and stable for a large class of memristor-based neural networks with time-varying delays. Moreover, a novel and useful finite-time complete synchronization condition is obtained in terms of linear matrix inequalities to ensure the synchronization goal in Ref.^36^.

In this work, we extend these studies and the mathematical model of MNN with mixed delays. In fact, we analyse the stability of equilibrium points with executing significant results of the period behavior of the system. After that, we study the phenomena of synchronization from the point of view of the theory and control. In the considered system, the weights are discontinuous; the classical definition of the solution for differential equations cannot apply here. Therefore we shall propose the Filippov solution to handle this problem. Filippov developed a solution to the differential equation with a discontinuous right-hand side^37^. Based on this definition, a differential equation with a discontinuous right-hand side has the same solution set as a differential inclusion. Our contribution consists to investigate the existence and exponential stability of the periodic solutions for memristor-based neural networks with time-varying delays in the leakage term. The stability properties of this model are then analyzed and we show that the solutions of this new linear system converge to a periodic and stable limit cycle. The main novelty of our contribution lies in solving the problems of stability and synchronization and we demonstrate the impact of the mixed delays. Also results enhance and extend earlier studies on neural network dynamical systems with a continuous or discontinuous right-hand side that are memristor-based or conventional.

The rest of this paper is organized as follows. A delayed memristor-based neural networks is presented and some necessary definitions are given in “Model description and preliminaries” section. In “Uniqueness and global exponential stability” section, we introduce the Filippov’s solution for our system and the existence of periodic solutions of the system. Our approaches are based on the differential inclusions and topological degree theories in set-valued analysis. In “Finite-time periodic synchronization” section, we shall study the uniqueness and global exponential stability of the w-periodic solution for the system. Especially, when the system is autonomous, we will give the sufficient conditions, uniqueness and global exponential stability of equilibrium point of the proposed system. Moreover, we designed novel control algorithms for the finite-time periodic synchronization to select neurons to pin the designed controller. In “Conclusions” section, a numerical example is obtained to show the effectiveness of the theoretical results given in the previous sections. It should be mentioned that the main results of this paper are Theorems 1–5.

## Model description and preliminaries

In this paper, we shall investigate the following memristive neural networks with time-varying delay:

1 $$\begin{aligned} \begin{array}{l} {\displaystyle \dot{x}_{i}(t) =-a_{i}(t)x_{i}(t)+\overset{n}{\underset{j=1}{\sum }}b_{ij}(x_{j}(t))f_{j}(x_{j}(t))+\overset{n}{\underset{j=1}{\sum }}c_{ij}(x_{j}(t-\tau _{ij}(t)))g_{j}(x_{j}(t-\tau _{ij}(t)))}\\ \quad \times {\displaystyle \overset{n}{\underset{j=1}{\sum }}p_{ij}(x_{j}(t))\overset{t}{\underset{-\infty }{\int }}k_{ij}(t-s)h_{j}(x_{j}(s))ds}+J_{i}(t), \end{array} \end{aligned}$$

where $n\ge 1$ represents the number of neurons in the network, $x_{i}(t)$ correponds to the potential membrane of the neuron *i* at time *t*, the $a_{i}$ is a positive constant rate with which the *i* th neuron will reset its potential to the resting state in isolation when it is disconnected. In addition, $f_{j},\,g_{j},\,h_{j}$ and $\phi _{j}$ denote the activation functions of *j*th neuron at time *t*, $b_{ij}(t),c_{ij}(t),$ $p_{ij}(t)$ are the synaptic connection weight of the unit *j* on the unit *i* at time *t*, $J_{i}$ is the input unit *i* and $\tau _{ij}(t)$ corresponds to the transmission delay of the ith unit along the axon of the jth unit at time t and is continuously differentiable function satisfying

2 $$\begin{aligned} \displaystyle 0\le \tau _{ij}(t)\le \tau , \end{aligned}$$

where $\tau =max_{1\le i,j\le n}\left\{ max_{t\in \left[ 0,\omega \right] },\tau _{ij}(t)\right\} ,$ $\tau$ is a nonnegative constant, $b_{ij}(t)$, $c_{ij}(t-\tau _{ij}(t))$ and $p_{ij}(t)$ are memristive synaptic weights. Basing on memristor feature and the current-voltage characteristic, we write:

3 $$\begin{aligned} \displaystyle b_{ij}(x_{i}(t))= & {} {\left\{ \begin{array}{ll} \bar{b}_{ij}, &{} |x_{j}(t)|>T_{j}\\ \displaystyle \underline{b}_{ij}, &{} |x_{j}(t)|<T_{j}\\ \end{array}\right. }, \end{aligned}$$

4 $$\begin{aligned} \displaystyle c_{ij}(x_{i}(t))= & {} {\left\{ \begin{array}{ll} \displaystyle \overline{c}_{ij}, &{} |x_{j}(t)|>T_{j}\\ \displaystyle \underline{c}_{ij}, &{} |x_{j}(t)|<T_{j} \end{array}\right. }, \end{aligned}$$

5 $$\begin{aligned} \displaystyle p_{ij}(x_{i}(t))= & {} {\left\{ \begin{array}{ll} \overline{p}_{ij}, &{} |x_{j}(t)|>T_{j}\\ \displaystyle \underline{p}_{ij}, &{} |x_{j}(t)|<T_{j} \end{array}\right. }, \end{aligned}$$

for $i,\,j=1,2,\ldots ,n$; $t\in R$, where $T_{j}>0$ is a switching jumps and let $\bar{a}_{i}>0$, $\underline{a}_{i}>0$, $\overline{b}_{ij}$, $\underline{b}_{ij}$ , $\overline{c}_{ij}$, $\underline{c}_{ij}$, $\overline{p}_{ij}$, $\underline{p}_{ij}$ for $i,j=1,2,\ldots ,n$ are all constants.

### Definition 1

*(Periodic solution)*. A solution *x*(*t*) of system (1) on $[0,+\infty [$ is a $\omega$-periodic solution with period $\omega$ if

$$x(t+\omega )=x(t)$$, for all $$t\ge 0$$.

Throughout this paper, we always assume the following hypothesis:

**(H1)** $a_{i}(.),\overline{b}_{ij}(.),\underline{b}_{ij}(.),\overline{c}_{ij}(.),\underline{c}_{ij}(.),\overline{p}_{ij}(.),\underline{p}_{ij}(.),\tau _{ij}(.)$ and $J_{i}(.)$ are continuous and w-periodic functions.

**(H2)** The neuron activation functions $f_{j}(.)$, $g_{j}(.)$ and $h_{j}(.)$ are Lipschitz-continuous on $\mathbb {R}$ with Lipschitz constants $F_{j}>0$, $G_{j}>0$ and $H_{j}>0$ respectively, i.e.,

$$\begin{array}{c} |f_{j}(x)-f_{j}(y)|<F_{j}|x-y|,|g_{j}(x)-g_{j}(y)|<G_{j}|x-y|, |h_{j}(x)-h_{j}(y)|<H_{j}|x-y|, \end{array}$$ for all $x,y\in \mathbb {R}$ and for all $j=1,2,..,n.$

**(H3)** $$\exists M,\alpha \in \mathbb {R}_{+}$$ such that $$|k_{ij}(t-s)|= {\left\{ \begin{array}{ll} =0, &{} s\le 0\\ \le Me^{-\alpha (t-s)}, &{} 0\le s\le t \end{array}\right. }.$$

### Definition 2

We say that a square matrix is an M-matrix if it has all nonpositive elements outside the diagonal and all positive successive principal minors^38^.

### Lemma 1

^39^ Given matrix $M=(m_{ij})_{n\times n}$ with nonpositive off-diagonal elements is a nonsingular M-matrix if and only if one of the following conditions holds:

1. There exist *n* positive constants $\alpha _{1},\alpha _{2},\ldots \alpha _{n}$ such that $\alpha _{i}m_{ii}+\overset{n}{\underset{j=1,j\ne i}{\sum }}\alpha _{j}m_{ji}>0,\,i=1,\ldots ,n.$
2. There exist *n* positive constants $\beta _{1},\beta _{2},\ldots \beta _{n}$ such that $\beta _{i}m_{ii}+\overset{n}{\underset{j=1,j\ne i}{\sum }}\beta _{j}m_{ij}>0,\,i=1,\ldots ,n$.

### Definition 3

*(Clarke Regular*^40^*)* $V(x):\mathbb {R}^{n}\rightarrow \mathbb {R}$ is said to be regular, if for each $x\in \mathbb {R}^{n}$ and $v\in \mathbb {R}^{n}$

1. there exists the usual right directional derivative $D^{+}V(x,v)=\underset{h\longrightarrow 0^{+}}{lim}\frac{V(x+hv)-V(x)}{h}$,
2. the generalized directional derivative of *V* at *x* in the direction $v\in \mathbb {R}^{n}$ is defined as $D^{++}V(x,v)=\underset{y\rightarrow x,h\longrightarrow 0^{+}}{lim}\frac{V(y+hv)-V(y)}{h}$, then $D^{+}V(x,v)=D^{++}V(x,v)$.

### Definition 4

Consider the column vector $x=(x_{1},x_{2},\ldots ,x_{n})^{T}$, where *T* denotes the transpose of a vector, $\left\| x\right\|$ denotes any vector norm in $\mathbb {R}^{n}$. $\left\| x\right\| _{1}=\overset{n}{\underset{j=1}{\sum }}|x_{i}|,$ $\left\| x\right\| _{2}=\left[ \overset{n}{\underset{j=1}{\sum }}x_{i}^{2}\right] ^{\frac{1}{2}}$.

Let the set $A\in \mathbb {R}^{n},\,\overline{co}[A]$ denotes the closure of the convex hull of *A*, $\mu (A)$ is the Lebesgue measure of *A*, and $\partial A$ is the boundary of *A*.

### Definition 5

For a locally Lipschitz function $V(x):\mathbb {R}^{n}\rightarrow \mathbb {R}$, we can define Clarke’s generalized gradient of *V* at point *x*, as follows

$$\partial V(x)=\overline{co}[\underset{k\leftarrow \infty }{lim}\nabla V(x_{k}):x_{k}\rightarrow x,\,x_{k}\notin N,\,x_{k}\notin \Omega ],$$

where $\Omega \subset \mathbb {R}^{n}$ is the set of points where *V* is not differentiable and $N\subset \mathbb {R}^{n}$ is an arbitrary set with measure zero^41^. In the following, for a continuous $\omega$-periodic function *f*(*t*) defined on $\mathbb {R}$, we define

$$\bar{f}=\frac{1}{\omega }\intop _{0}^{\omega }f(t)dt,\,f^{u}=\underset{t\in \left[ o,\omega \right] }{sup}|f(t)|,\,f^{l}=\underset{t\in \left[ o,\omega \right] }{inf}|f(t)|.$$

Given $C_{\tau }:=C([-\tau ,0])$ defines a Banach space of all continuous functions $e:[-\tau ,0]\rightarrow \mathbb {R}$.

For $x\in \mathbb {R}^{n}$, we can write $x\in C_{\tau }$ means $x(s)\equiv x$ in $[-\tau ,0]$. Given $e\in C_{\tau }$, let $\left\| e\right\| _{c}=sup\left| e(s)\right|$.

The initial states proposed for system (1) are as follow

6 $$\begin{aligned} \displaystyle x_{i}(s)=e_{i}(s),\,s\in [-\tau ,0],\,i=1,2,\ldots ,n. \end{aligned}$$

Consider $x_{t}\in C([-\tau ,0],\mathbb {R}^{n})$ described by $x_{t}(s)=x(t+s),$ $-\tau \le s\le 0$, and the initial states (10) can be rewritten as

7 $$\begin{aligned} \displaystyle x_{0}=e\in C_{\tau }:=C([-\tau ,0],\mathbb {R}^{n}). \end{aligned}$$

Suppose that $A\subset \mathbb {R}^{n}$, then $x\rightarrow \phi (x)$ is said a set-valued map from *A* to $\mathbb {R}^{n}$, if for every point $x\in A$, there exists a nonempty set $\phi (x)\subset \mathbb {R}^{n}$. We call a set-valued map $\phi$ with nonempty values, an upper semicontinuous at $x_{0}\in A$, if for every open set *N* containing $\phi (x_{0})$, there exists a neighborhood *M* of $x_{0}$ such that $\phi (M)\subset N$. The map $\phi (x)$ is said to have a closed (convex, compact) image if for each $x\in A$, $\phi (x)$ is closed (convex, compact).

## Existence of periodic solution

In the rest of this section we will investigate the existence of periodic solutions of the generalized memristor system.

By the differential equation system (1), we consider the set-valued maps as follow: for $t\in \mathbb {R}$ and $i,j=1,2,\ldots ,n,$

8 $$\begin{aligned} \displaystyle K[b_{ij}(x_{j}(t))]= & {} {\left\{ \begin{array}{ll} \bar{b}_{ij}, &{} |x_{j}(t)|>T_{j}\\ \displaystyle \overline{co}\left\{ \bar{b}_{ij},\underline{b}_{ij}\right\} , &{} |x_{i}(t)|=T_{i}\\ \displaystyle \underline{b}_{ij}, &{} |x_{j}(t)|<T_{j} \end{array}\right. }, \end{aligned}$$

9 $$\begin{aligned} \displaystyle K[c_{ij}(x_{j}(t))]= & {} {\left\{ \begin{array}{ll} \overline{c}_{ij}, &{} |x_{j}(t)|>T_{j}\\ \displaystyle \overline{co}\left\{ \bar{c}_{ij},\underline{c}_{ij}\right\} , &{} |x_{j}(t)|=T_{j}\\ \displaystyle \underline{c}_{ij}, &{} |x_{j}(t)|<T_{j} \end{array}\right. }, \end{aligned}$$

10 $$\begin{aligned} \displaystyle K[p_{ij}(x_{j}(t))]= & {} {\left\{ \begin{array}{ll} \overline{p}_{ij}, &{} |x_{j}(t)|>T_{j}\\ \displaystyle \overline{co}\left\{ \bar{p}_{ij},\underline{p}_{ij}\right\} , &{} |x_{j}(t)|=T_{j}\\ \displaystyle \underline{p}_{ij}, &{} |x_{i}(t)|<T_{i} \end{array}\right. }. \end{aligned}$$

It is clear that $K[b_{ij}(x_{i}(t))]$, $K[c_{ij}(x_{i}(t))]$ and $K[p_{ij}(x_{i}(t))]$ are all closed, convex and compact for every $t\in \mathbb {R}$ and $i,j=1,\ldots ,n$.

A function *x*(*t*) is said to be a solution of system (1) on [0, *T*) with initial condition (7) or (8), if *x*(*t*) is absolutely continuous on any compact interval of [0, *T*] and satisfies differential inclusions:

11 $$\begin{aligned} \begin{array}{l} \displaystyle \frac{dx_{i}(t)}{dt}\in -a_{i}(t)x_{i}(t)+\overset{n}{\underset{j=1}{\sum }}K[b_{ij}(x_{j}(t))]f_{j}\left( x_{j}(t)\right) \\ \quad \displaystyle +\overset{n}{\underset{j=1}{\sum }}K[c_{ij}(x_{j}(t))]g_{j}\left( x_{j}\left( t-\tau _{ij}(t)\right) \right) +\overset{n}{\underset{j=1}{\sum }}K[p_{ij}(x_{j}(t))]\overset{t}{\underset{-\infty }{\int }}k_{ij}(t-s)h_{j}\left( x_{j}(s)\right) ds+J_{i}(t), \end{array} \end{aligned}$$

or there exist $\gamma _{ij}(t)\in K[b_{ij}(x_{i}(t))]$, $\eta _{ij}(t)\in K[c_{ij}(x_{i}(t))]$ and $\nu _{ij}(t)\in K[p_{ij}(x_{i}(t))]$ satisfy

12 $$\begin{aligned} \begin{array}{c} \displaystyle \frac{dx_{i}(t)}{dt}\in -a_{i}(t)x_{i}(t)+\overset{n}{\underset{j=1}{\sum }}\gamma _{ij}(t)f_{j}\left( x_{j}(t)\right) +\overset{n}{\underset{j=1}{\sum }}\eta _{ij}(t)g_{j}\left( x_{j}\left( t-\tau \right) \right) +\overset{n}{\underset{j=1}{\sum }}\nu _{ij}(t)\overset{t}{\underset{-\infty }{\int }}k_{ij}(t-s)h_{j}\left( x_{j}\right) ds+J_{i}, \end{array} \end{aligned}$$

for a. a. $$t\in [0,T),\,i=1,2,\ldots ,n.$$

In the following, we discuss dynamical behavior of system (1) using the following set-valued version of the Mawhin coincidence theorem.

### Lemma 2

(Mawhin coincidence theorem^42^) Suppose that $\phi :\mathbb {R}\times \mathbb {R}^{n}\rightarrow K_{\nu }(\mathbb {R}^{n})$ is upper semicontinuous and $\omega$-periodic in *t*. If the following conditions hold:

1. There exists a bounded open set $\Delta \subset C_{\omega }$, the set of all continuous, $\omega$-periodic functions: $\mathbb {R}\rightarrow \mathbb {R}^{n}$, such that for any $\lambda \in (0,1)$, each $\omega$-periodic function *x*(*t*) of the inclusion
  13 $$\begin{aligned} \displaystyle \frac{dx}{dt}\in \lambda \phi (t,x), \end{aligned}$$
  satisfies $x\notin \partial \Delta$ if it exists.
2. Each solution $z\in \mathbb {R}^{n}$ of the inclusion $0\in \frac{1}{\omega }\intop _{0}^{\omega }\phi (t,z)dt=g_{0}(z)$ satisfies $z\notin \partial \Delta \cap \mathbb {R}^{n};$
3. $deg(g_{0},\Delta \cap \mathbb {R}^{n},0)\ne 0$, then differential inclusion (13) has at least one $\omega$-periodic solution *x*(*t*) with $x\in \bar{\Delta }$. If a matrix $O\ge 0$ then all elements of *O* are greater than or equal to 0, and similarly we can describe $O>0$. It follows that for given vectors or matrices *O* and *P*, $O\ge P$ (or $O>P$) and similarly that $O-P\ge 0$ (or $O-P>0$). After that, we give sufficient conditions to guarantee the existence of periodic solutions for the memristive neural network.

### Theorem 1

We consider $I\rightarrow S$ an M-matrix, where *I* is the identity matrix of dimension *n*, $S=(s_{ij})_{n\times n}$ and

**(H4)** $$s_{ij}=\frac{1}{a_{i}^{l}}\left( b_{ij}^{u}F_{j}+\frac{c_{ij}^{u}G_{j}}{\sqrt{1-\tau _{ij}^{D}}}+\frac{M}{\alpha }p_{ij}^{u}F_{j}\right) ,\,i,\,j=1,2,..,n,$$

where $$b_{ij}^{u}=max\left\{ \overline{b}_{ij}^{u},\underline{b}_{ij}^{u}\right\} ,$$ $$c_{ij}^{u}=max\left\{ \overline{c}_{ij}^{u},\underline{c}_{ij}^{u}\right\}$$ and $$p_{ij}^{u}=max\left\{ \overline{p}_{ij}^{u},\underline{p}_{ij}^{u}\right\} .$$

Then there exists at least one $\omega$-periodic solution of system (1).

### Proof

Define $E_{\omega }=\left\{ x(t)\in C(\mathbb {R},\mathbb {R}^{n}):\,x(t+\omega )=x(t)\right\} ,$ and for $x(t)\in E_{\omega }$

$$\left\| x(t)\right\| _{C_{\omega }}=\overset{n}{\underset{i=1}{\sum }}\underset{t\in \left[ 0,\omega \right] }{max}|x_{i}(t)|.$$

Then $E_{\omega }$ is a Banach space equipped with the norm $\left\| .\right\| _{E_{\omega }}$.

Let for $$x(t)\in E_{\omega }$$,

$$\begin{aligned} \phi (t,x)=(\phi _{1}(t,x),\phi _{2}(t,x),\ldots ,\phi _{n}(t,x))^{T}, \end{aligned}$$

where

$$\begin{aligned} \phi _{i}(t,x){} & {} = -a_{i}(t)x_{i}(t)+\overset{n}{\underset{j=1}{\sum }}K[b_{ij}(x_{j}(t))]f_{j}\left( x_{j}(t)\right) +\overset{n}{\underset{j=1}{\sum }}K[c_{ij}(x_{j}(t))]f_{j}\left( x_{j}\left( t-\tau \right) \right) \\{} & {} \quad +\overset{n}{\underset{j=1}{\sum }}K[p_{ij}(x_{j}(t))]\overset{t}{\underset{-\infty }{\int }}f_{j}\left( x_{j}(s)\right) ds+J_{i}(t), \end{aligned}$$

$$i=1,2,..,n.$$

Let assumption (H4) holds, $\phi (t,x)$ is an upper semicontinuous set-valued map with nonempty compact convex values under H4. Here we need to find an appropriate open, bounded subset $\Delta$, in order to apply Mawhin-Like Coincidence Theorem (Lemma 2),

From the differential inclusion (13), we obtain

14 $$\begin{aligned} \begin{array}{l}\displaystyle \frac{dx_{i}(t)}{dt}\in \lambda [-a_{i}x_{i}(t)+\overset{n}{\underset{j=1}{\sum }}K[b_{ij}(x_{j}(t))]f_{j}\left( x_{j}(t)\right) +\overset{n}{\underset{j=1}{\sum }}K[c_{ij}(x_{j}(t))]f_{j}\left( x_{j}\left( t-\tau \right) \right) \\ \quad +\overset{n}{\underset{j=1}{\sum }}K[p_{ij}(x_{j}(t))]\overset{t}{\underset{-\infty }{\int }}f_{j}\left( x_{j}(s)\right) ds+J_{i}]. \end{array} \end{aligned}$$

Given $x(t)=(x_{1}(t),x_{2}(t),\ldots ,x_{n}(t))^{T}$ an arbitrary $\omega$-periodic solution of the differential inclusion (14) for a certain $\lambda \in (0,1)$. There exist $\gamma _{ij}(t)\in K[b_{ij}(x_{j}(t))]$ and $\eta _{ij}(t)\in K[c_{ij}(x_{j}(t))]$ $\nu _{ij}(t)\in K[p_{ij}(x_{j}(t))]$ satisfy

15 $$\begin{aligned} \begin{array}{ll} \displaystyle \frac{dx_{i}(t)}{dt}=\lambda [-a_{i}(t)x_{i}(t)+\overset{n}{\underset{j=1}{\sum }}\gamma _{ij}(t)f_{j}\left( x_{j}(t)\right) +\overset{n}{\underset{j=1}{\sum }}\eta _{ij} (t)f_{j}\left( x_{j}\left( t-\tau \right) \right) +\overset{n}{\underset{j=1}{\sum }}\nu _{ij}(t)\overset{t}{\underset{-\infty }{\int }}f_{j}\left( x_{j}(s)\right) ds+J_{i}], \end{array} \end{aligned}$$

for a [0, *T*),  $$i=1,2,..,n.$$

Multiplying both sides of (15) by $x_{i}(t)$ and integrating over the interval $[0,\omega ]$, we get

16 $$\begin{aligned} \begin{array}{l} \displaystyle \intop _{0}^{\omega }a_{i}(t)x_{i}^{2}(t)dt =\intop _{0}^{\omega }x_{i}(t)[\overset{n}{\underset{j=1}{\sum }}\gamma _{ij}(t)f_{j}\left( x_{j}(t)\right) +\overset{n}{\underset{j=1}{\sum }}\eta _{ij}(t)g_{j}\left( x_{j}\left( t-\tau _{ij}\right) \right) \\ \quad +\overset{n}{\underset{j=1}{\sum }}\nu _{ij}(t)\overset{t}{\underset{-\infty }{\int }}k_{ij}(t-s)h_{j}\left( x_{j}(s)\right) ds+J_{i}(t)]dt. \end{array} \end{aligned}$$

From (H2), (8), (9), (10) and (11), it is clear to see that

17 $$\begin{aligned} \begin{array}{c} \displaystyle {\left\{ \begin{array}{ll} |\gamma _{ij}(t)|&{} \le max\left\{ |\overline{b}_{ij}|,|\underline{b}_{ij}|\right\} \le b_{ij}^{u}\\ \displaystyle |\eta _{ij}(t)|&{} \le max\left\{ |\overline{c}_{ij}|,|\underline{c}_{ij}|\right\} \le c_{ij}^{u}\\ \displaystyle |\nu _{ij}(t)|&{} \le max\left\{ |\overline{p}_{ij}|,|\underline{p}_{ij}|\right\} \le p_{ij}^{u}\\ \displaystyle |f_{j}(x_{j}(t))|&{} \le F_{j}|x_{j}(t)|+|f_{j}(0)|,\\ |g_{j}(x_{j}(t-\tau _{ij}))|&{} \le G_{j}|x_{j}(t-\tau _{ij})|+|g_{j}(0)|\\ \displaystyle |h_{j}(x_{j}(t))|&{} \le H_{j}|x_{j}(t)|+|h_{j}(0)|\end{array}\right. }.\end{array} \end{aligned}$$

From (16) and Cauchy–Schwarz inequality, we obtain:

18 $$\begin{aligned} \begin{array}{l} \displaystyle a_{i}^{l}\intop _{0}^{\omega }x_{i}^{2}(t)dt\le \overset{n}{\underset{j=1}{\sum }}b_{ij}^{u}F_{j}\intop _{0}^{\omega }|x_{i}(t)||x_{j}(t)|dt+\overset{n}{\underset{j=1}{\sum }}c_{ij}^{u}G_{j}\intop _{0}^{\omega }|x_{i}(t)||x_{j}(t-\tau _{ij})|dt\\ \quad +\overset{n}{\underset{j=1}{\sum }}p_{ij}^{u}H_{j}\intop _{0}^{\omega }\left( \overset{t}{\underset{-\infty }{\int }}k_{ij}(t-s)|x_{i}(s)||x_{j}(s)|ds\right) dt\\ \displaystyle \quad +\left( \overset{n}{\underset{j=1}{\sum }}b_{ij}^{u}|f_{j}(0)|+c_{ij}^{u}|g_{j}(0)|+\frac{M}{\alpha }p_{ij}^{u}|h_{j}(0)|+J_{i}^{I}\right) \intop _{0}^{\omega }|x_{i}(t)|dt. \end{array} \end{aligned}$$

Noticing that

19 $$\begin{aligned} \intop _{0}^{\omega }|x_{j}^{2}(t-\tau _{ij}(t))|dt= & {} \intop _{-\tau _{ij}(0)}^{\omega -\tau _{ij}(\omega )}\frac{|x_{j}^{2}(t)|}{1-\dot{\tau }_{ij}(\varphi _{ij}^{-1}(t))}dt =\intop _{-\tau _{ij}(0)}^{\omega -\tau _{ij}(0)}\frac{|x_{j}^{2}(t)|}{1-\dot{\tau }_{ij}(\varphi _{ij}^{-1}(t))}dt=\intop _{0}^{\omega }\frac{|x_{j}^{2}(t)|}{1-\dot{\tau }_{ij}(\varphi _{ij}^{-1}(t))}dt\nonumber \\ \displaystyle= & {} \frac{1}{1-\tau _{ij}^{D}}\intop _{0}^{\omega }|x_{j}^{2}(t)|dt,\,\,j=1,2,..,n, \end{aligned}$$

where $\varphi _{ij}^{-1}$ is the inverse function of $\varphi _{ij}=t-\tau _{ij}(t),\,i,j=1,2,..,n$.

Then, for i=1,2,..,n, we obtain

20 $$\begin{aligned} \displaystyle a_{i}^{l}\intop _{0}^{\omega }x_{i}^{2}(t)dt\le & {} \overset{n}{\underset{j=1}{\sum }}\left( b_{ij}^{u}F_{j}+\frac{c_{ij}^{u}G_{j}}{\sqrt{1-\tau _{ij}^{D}}}+\frac{M}{\alpha }p_{ij}^{u}H_{j}\right) \left( \intop _{0}^{\omega }|x_{j}^{2}(t)|dt\right) {}^{\frac{1}{2}} +\sqrt{\omega }\left( \overset{n}{\underset{j=1}{\sum }}b_{ij}^{u}|f_{j}(0)|+c_{ij}^{u}|g_{j}(0)|\right. \nonumber \\{} & {} \left. +\frac{M}{\alpha }p_{ij}^{u}|h_{j}(0)|+J_{i}^{I}\right) , \end{aligned}$$

which implies

21 $$\begin{aligned} \intop _{0}^{\omega }x_{i}^{2}(t)dt\le & {} \overset{n}{\underset{j=1}{\sum }}\frac{1}{a_{i}^{l}}\left( b_{ij}^{u}F_{j}+\frac{c_{ij}^{u}G_{j}}{\sqrt{1-\tau _{ij}^{D}}}+\frac{M}{\alpha }p_{ij}^{u}H_{j}\right) \left( \intop _{0}^{\omega }|x_{j}^{2}(t)|dt\right) {}^{\frac{1}{2}}\nonumber \\{} & {} +\frac{\sqrt{\omega }}{a_{i}^{l}}\left( \overset{n}{\underset{j=1}{\sum }}b_{ij}^{u}|f_{j}(0)|+c_{ij}^{u}|g_{j}(0)|+\frac{M}{\alpha }p_{ij}^{u}|h_{j}(0)|+J_{i}^{I}\right) \nonumber \\{} & {} \displaystyle \intop _{0}^{\omega }x_{i}^{2}(t)dt\triangleq q_{ij}\overset{n}{\underset{j=1}{\sum }}\left( \intop _{0}^{\omega }x_{j}^{2}(t)dt\right) ^{\frac{1}{2}}+\sqrt{\omega }\theta _{i}\,i=1,2,..,n, \end{aligned}$$

where

$$\displaystyle \theta _{i}=\frac{1}{a_{i}^{l}}\left( \overset{n}{\underset{j=1}{\sum }}b_{ij}^{u}|f_{j}(0)|+c_{ij}^{u}|g_{j}(0)|+\frac{M}{\alpha }p_{ij}^{u}|h_{j}(0)|+J_{i}^{I}\right) ,\,i=1,2,..,n.$$

Let $$\left\| x_{i}\right\| _{2}^{\omega }=\left( \intop _{0}^{\omega }x_{i}^{2}(t)dt\right) ^{2},\,x_{i}\in C\left( \mathbb {R},\mathbb {R}\right) ,\,i=1,2,..,n.$$

Thus

22 $$\begin{aligned} \displaystyle \left( I-S\right) \left( \left\| x_{1}\right\| _{2}^{\omega },\left\| x_{2}\right\| _{2}^{\omega },\ldots ,\left\| x_{n}\right\| _{2}^{\omega }\right) ^{T}\le \sqrt{\omega }\alpha . \end{aligned}$$

Since $\left( I-S\right)$ is an M-matrix, assumption (H4) holds and Lemma 1, there exists a vector

23 $$\begin{aligned} \displaystyle \nu =\left( \nu _{1}^{*},\nu _{2}^{*},\ldots ,\nu _{n}^{*}\right) =\nu \left( I-S\right) >\left( 0,0,\ldots ,0\right) , \end{aligned}$$

and from (22), we have

24 $$\begin{aligned}{} & {} min\left\{ \nu _{1}^{*},\nu _{2}^{*},\ldots ,\nu _{n}^{*}\right\} \left( \left\| x_{1}\right\| _{2}^{\omega },\left\| x_{2}\right\| _{2}^{\omega },\ldots ,\left\| x_{n}\right\| _{2}^{\omega }\right) \le \nu _{1}^{*}\left\| x_{1}\right\| _{2}^{\omega }+\nu _{2}^{*}\left\| x_{2}\right\| _{2}^{\omega }+\ldots +\nu _{n}^{*}\left\| x_{n}\right\| _{2}^{\omega }\nonumber \\{} & {} \quad =\nu \left( I-S\right) \left( \left\| x_{1}\right\| _{2}^{\omega },\left\| x_{2}\right\| _{2}^{\omega },\ldots ,\left\| x_{n}\right\| _{2}^{\omega }\right) ^{T} \le \nu \sqrt{\omega }\left( \theta _{1},\theta _{2},\ldots ,\theta _{n}\right) ^{T}\nonumber \\{} & {} \quad \displaystyle =\sqrt{\omega }\overset{n}{\underset{j=1}{\sum }}\nu _{i}\theta _{i}. \end{aligned}$$

Thus, we obtain

25 $$\begin{aligned} \displaystyle \left( \intop _{0}^{\omega }|x_{i}^{2}(t)|dt\right) {}^{\frac{1}{2}}=\left\| x_{i}\right\| _{2}^{\omega }\le \frac{\sqrt{\omega }\overset{n}{\underset{i=1}{\sum }}\nu _{i}\theta _{i}}{min\left\{ \nu _{1}^{*},\nu _{2}^{*},\ldots ,\nu _{n}^{*}\right\} }\triangleq \sqrt{\omega }N. \end{aligned}$$

Obviously, we can see that there exists $t_{i}\in \left[ 0,\omega \right]$ such that

26 $$\begin{aligned} \displaystyle |x_{i}\left( t\right) |\le \sqrt{\omega }N,\,i=1,2,\ldots ,n. \end{aligned}$$

Since for $$t_{i}\in \left[ 0,\omega \right]$$,

$$\begin{aligned} x_{i}(t)=x_{i}(t_{i})+\intop _{t_{i}}^{t}\dot{x}_{i}\left( s\right) ds, \end{aligned}$$

thus we obtain

$$\begin{aligned} |x_{i}(t)|\le N+\intop _{0}^{\omega }\dot{x}_{i}\left( s\right) ds,\,i=1,2,..,n, \end{aligned}$$

we can derive from (17) and (19) that

$$\begin{aligned}{} & {} \intop _{0}^{\omega }|\dot{x}_{i}(t)|dt<a_{i}^{l}\intop _{0}^{\omega }|x_{i}(t)|dt+\overset{n}{\underset{j=1}{\sum }}b_{ij}^{u}F_{j}\left( \intop _{0}^{\omega }|x_{j}(t)|dt\right) \\{} & {} \qquad +\overset{n}{\underset{j=1}{\sum }}c_{ij}^{u}G_{j}\intop _{0}^{\omega }|x_{j}(t-\tau _{ij}(t))|dt+\overset{n}{\underset{j=1}{\sum }}p_{ij}^{u}H_{j}\left( \intop _{0}^{\omega }\overset{t}{\underset{-\infty }{\int }}k_{ij}(t-s)|x_{j}(s)|dsdt\right) \\{} & {} \qquad +\omega \left( \overset{n}{\underset{j=1}{\sum }}\left( b_{ij}^{u}|f_{j}(0)|+c_{ij}^{u}|g_{j}(0)|+\frac{M}{\alpha }p_{ij}^{u}|h_{j}(0)|\right) +J_{i}^{I}\right) \\{} & {} \quad = \intop _{0}^{\omega }|\dot{x}_{i}(t)|dt<a_{i}^{l}\intop _{0}^{\omega }|x_{i}(t)|dt+\overset{n}{\underset{j=1}{\sum }}b_{ij}^{u}F_{j}\left( \intop _{0}^{\omega }|x_{j}(t)|dt\right) +\overset{n}{\underset{j=1}{\sum }}c_{ij}^{u}G_{j}\intop _{0}^{\omega }|x_{j}(t-\tau _{ij}(t))|dt\\{} & {} \qquad +\overset{n}{\underset{j=1}{\sum }}p_{ij}^{u}H_{j}\left( \intop _{-\infty }^{\omega }|x_{j}(s)|\overset{\omega }{\underset{s}{\int }}k_{ij}(t-s)dtds\right) +\omega \left( \overset{n}{\underset{j=1}{\sum }}\left( b_{ij}^{u}|f_{j}(0)|+c_{ij}^{u}|g_{j}(0)|\right) +\frac{M}{\alpha }p_{ij}^{u}|h_{j}(0)|+J_{i}^{I}\right) \\{} & {} \quad = \intop _{0}^{\omega }|\dot{x}_{i}(t)|dt<a_{i}^{l}\intop _{0}^{\omega }|x_{i}(t)|dt+\overset{n}{\underset{j=1}{\sum }}b_{ij}^{u}F_{j}\left( \intop _{0}^{\omega }|x_{j}(t)|dt\right) +\overset{n}{\underset{j=1}{\sum }}c_{ij}^{u}G_{j}\intop _{0}^{\omega }|x_{j}(t-\tau _{ij}(t))|dt\\{} & {} \qquad +\overset{n}{\underset{j=1}{\sum }}p_{ij}^{u}H_{j}\left( \intop _{0}^{\omega }|x_{i}(s)|\left( \overset{\omega }{\underset{s}{\int }}k_{ij}(t-s)dt\right) ds\right) +\omega \left( \overset{n}{\underset{j=1}{\sum }}\left( b_{ij}^{u}|f_{j}(0)|+c_{ij}^{u}|g_{j}(0)|\right) +\frac{M}{\alpha }p_{ij}^{u}|h_{j}(0)|+J_{i}^{I}\right) \\{} & {} \quad \le \intop _{0}^{\omega }|\dot{x}_{i}(t)|dt<a_{i}^{l}\intop _{0}^{\omega }|x_{i}(t)|dt+\overset{n}{\underset{j=1}{\sum }}b_{ij}^{u}F_{j}\left( \intop _{0}^{\omega }|x_{i}(t)|dt\right) +\overset{n}{\underset{j=1}{\sum }}c_{ij}^{u}G_{j}\intop _{0}^{\omega }|x_{j}(t-\tau _{ij}(t))|dt\\{} & {} \qquad +\overset{n}{\underset{j=1}{\sum }}\frac{M}{\alpha }p_{ij}^{u}H_{j}\left( \intop _{0}^{\omega }|x_{i}(s)|ds\right) +\omega \left( \overset{n}{\underset{j=1}{\sum }}\left( b_{ij}^{u}|f_{j}(0)|+c_{ij}^{u}|g_{j}(0)|\right) +\frac{M}{\alpha }p_{ij}^{u}|h_{j}(0)|+J_{i}^{I}\right) \\{} & {} \quad \le a_{i}^{l}\sqrt{\omega }\left\| x_{i}\right\| _{2}^{\omega }+\overset{n}{\underset{j=1}{\sum }}\left( b_{ij}^{u}F_{j}+\frac{c_{ij}^{u}G_{j}}{\sqrt{1-\tau _{ij}^{D}}}+\frac{M}{\alpha }p_{ij}^{u}H_{j}\right) \sqrt{\omega }\left\| x_{i}\right\| _{2}^{\omega }+\omega \left( \overset{n}{\underset{j=1}{\sum }}\left( b_{ij}^{u}|f_{j}(0)|+c_{ij}^{u}|g_{j}(0)|\right) +\frac{M}{\alpha }p_{ij}^{u}|h_{j}(0)|+J_{i}^{I}\right) \\{} & {} \quad \le \sqrt{\omega }N+\left( a_{i}^{l}+\overset{n}{\underset{j=1}{\sum }}\left( b_{ij}^{u}F_{j}+\frac{c_{ij}^{u}G_{j}}{\sqrt{1-\tau _{ij}^{D}}}+\frac{M}{\alpha }p_{ij}^{u}H_{j}\right) \right) +\omega \left( \overset{n}{\underset{j=1}{\sum }}\left( b_{ij}^{u}|f_{j}(0)|+c_{ij}^{u}|g_{j}(0)|\right) +\frac{M}{\alpha }p_{ij}^{u}|h_{j}(0)|+J_{i}^{I}\right) \triangleq R_{i}, \end{aligned}$$

for each $\,i=1,2,..,n.$ Then, it follows

27 $$\begin{aligned} \displaystyle |x_{i}(t)|\le N+R_{i}\triangleq H_{i},\,i=1,2,..,n. \end{aligned}$$

One may readily verify that $H_{i},\,i=1,2,..,n$ is independent of $\lambda$. Again taking (H4) into account, we can get from Lemma 1, that there exists a vector $\zeta =\left( \zeta _{1},\zeta _{2},\ldots ,\zeta _{n}\right) ^{T}>\left( 0,0,\ldots ,0\right) ^{T},$ such that $\left( I-S\right) \zeta >\left( 0,0,\ldots ,0\right) ^{T}$. Hence, we can choose a sufficiently large constant $\sigma$ such that

$$\zeta ^{*}=\left( \zeta _{1}^{*},\zeta _{2}^{*},\ldots ,\zeta _{n}^{*}\right) =\left( \sigma \zeta _{1},\sigma \zeta _{2},\ldots ,\sigma \zeta _{n}\right) ^{T}>\sigma \zeta ,$$ and that

28 $$\begin{aligned} \displaystyle \zeta _{i}^{*}=\sigma \zeta _{i}>H_{i}(i=1,2,\ldots ,n),\,and\left( I-S\right) \zeta ^{*}>\theta . \end{aligned}$$

In order to finish our proof we will proceed in three steps:

*Step1 * let us take

29 $$\begin{aligned} \displaystyle \Delta =\left\{ x(t)\in C_{\omega }:-\zeta ^{*}<x(t)<\zeta ^{*},\forall t\in \mathbb {R}\right\} . \end{aligned}$$

Hence $\Delta$ is an open bounded set of $C_{\omega }$ and for any $\lambda \in \left( 0,1\right)$, $x\notin \partial \Delta$ . Thus, the condition (1) in Mawhin coincidence theorem is satisfied.

*Step 2* we shall use contradiction to demonstrate the condition (2) in Lemma 2. Let us consider that there exists a solution $u=\left( u_{1},u,\ldots ,u_{n}\right) ^{T}\in \partial \Delta \cap \mathbb {R}^{n}$ of the inclusion

$$\begin{aligned} 0\in \frac{1}{\omega }\intop _{0}^{\omega }F(t,u)dt=g_{0}(u). \end{aligned}$$

Then *u* is a constant vector on $\mathbb {R}^{n}$ such that $|u_{i}|=\zeta _{i}$ for $i\in \left( 1,2,..,n\right)$.

Therefore, we have for $1<i\le n$

30 $$\begin{aligned} 0\in g_{0}(u){} & {} =-u_{i}\frac{1}{\omega }\intop _{0}^{\omega }a_{i}(t)u_{i}(t)]dt+\overset{n}{\underset{j=1}{\sum }}f_{j}(u)\frac{1}{\omega }\intop _{0}^{\omega }K[b_{ij}(u_{j}(t))]dt \nonumber \\{} & {} \quad +\overset{n}{\underset{j=1}{\sum }}g_{j}(u)\frac{1}{\omega }\intop _{0}^{\omega }K[c_{ij}(u_{j}(t))]dt +\overset{n}{\underset{j=1}{\sum }}\frac{1}{\omega }h_{j}(u)\intop _{0}^{\omega }K[b_{ij}(u_{j}(t))]dt\overset{t}{\underset{-\infty }{\int }}k_{ij}(t-s)ds+\frac{1}{\omega }\intop _{0}^{\omega }I_{i}(t)dt, \end{aligned}$$

or

31 $$\begin{aligned} 0{} & {} =-u_{i}\frac{1}{\omega }\intop _{0}^{\omega }a_{i}(t)dt+\overset{n}{\underset{j=1}{\sum }}f_{j}(u)\frac{1}{\omega }\intop _{0}^{\omega }\gamma _{ij}(t)dt+\overset{n}{\underset{j=1}{\sum }}g_{j}(u)\frac{1}{\omega }\intop _{0}^{\omega }\eta _{ij}(t))dt\nonumber \\{} & {} \quad +\overset{n}{\underset{j=1}{\sum }}\frac{M}{\alpha }h_{j}(u)\frac{1}{\omega }\intop _{0}^{\omega }\nu _{ij}(t)dt+\frac{1}{\omega }\intop _{0}^{\omega }I_{i}(t)dt,\,i=1,2,..,n, \end{aligned}$$

where $\gamma _{ij}(t)\in K[b_{ij}(u_{i})]$, $\eta _{ij}(t)\in K[c_{ij}(u_{i})]$ and $\nu _{ij}(t)\in K[p_{ij}(u_{i})]$. Then, there exists some $t^{*}\in [0,\omega )$ such that

32 $$\begin{aligned} \displaystyle -u_{i}a_{i}(t^{*})+\overset{n}{\underset{j=1}{\sum }}(\gamma _{ij}(t^{*})f_{j}(u) +\eta _{ij}(t^{*})g_{j}(u)+\nu _{ij}(t^{*})\frac{M}{\alpha }h_{j}(u))+I_{i}(t^{*})=0,\,i=1,2,..,n. \end{aligned}$$

It follows

33 $$\begin{aligned} \zeta _{i}^{*}{} & {} =|u_{i}|=\frac{1}{a_{i}(t^{*})}[\overset{n}{\underset{j=1}{\sum }}\left( \gamma _{ij}(t^{*})f_{j}(u)+\eta _{ij}(t^{*})g_{j}(u)+\nu _{ij}(t^{*})\frac{M}{\alpha }h_{j}(u)\right) +I_{i}(t^{*})] \nonumber \\{} & {} \le \frac{1}{a_{i}^{l}}[\overset{n}{\underset{j=1}{\sum }}(b_{ij}^{u}(F_{j}(|u_{j}|)+|f_{j}(0)|)+c_{ij}^{u}\left( G_{j}(|u_{j}|)+|g_{j}(0)|\right) +\frac{M}{\alpha }p_{ij}^{u}\left( H_{j}(|u_{j}|)+|h_{j}(0)|\right) )+I_{i}^{u},\nonumber \\ \displaystyle{} & {} \le \frac{1}{a_{i}^{l}}[\overset{n}{\underset{j=1}{\sum }}(b_{ij}^{u}\left( F_{j}(|u_{j}|)+|f_{j}(0)|\right) +\frac{c_{ij}^{u}\left( G_{j}(|u_{j}|)+|g_{j}(0)|\right) }{\sqrt{1-\tau _{ij}^{D}}}+\frac{M}{\alpha }p_{ij}^{u}\left( H_{j}(|u_{j}|)+|h_{j}(0)|\right) )+I_{i}^{u}]\\{} & {} = \overset{n}{\underset{j=1}{\sum }}s_{ij}|u_{j}|+\theta _{i} =\overset{n}{\underset{j=1}{\sum }}s_{ij}\zeta _{j}^{*}+\theta _{i}.\nonumber \end{aligned}$$

Therefore $(I-S)\zeta ^{*}\le \theta$, which contradicts the fact $(I-S)\zeta ^{*}>\theta$ and the condition 2 of Lemma 2 holds.

*Step 3* In order to prove condition 3 let us define homotopic set-valued map

$$\phi :\Delta \cap \mathbb {R}^{n}\times \left[ 0,1\right] \rightarrow A_{\omega }$$

$$(u,h)\longmapsto hdiag\left( -\bar{a}_{1},-\bar{a}_{2},\ldots ,-\bar{a}_{n}\right) u+\left( 1-h\right) g_{0}(u),$$

where

$$\bar{a}_{i}=\frac{1}{\omega }\intop _{0}^{\omega }a_{i}(t)dt,\,i=1,2,\ldots ,n.$$

if $u=\left( u_{1},u_{2},\ldots ,u_{n}\right) ^{T}\in \partial \Delta \cap \mathbb {R}^{n}$ then *u* is a constant vector on $\mathbb {R}^{n}$ such that $|u_{i}|=\zeta _{i}^{*}$ for some $i\in \left\{ 1,2,\ldots ,n\right\}$.

It follows that

34 $$\begin{aligned} \begin{array}{cc} \displaystyle \left( \phi \left( u,h\right) \right) _{i}=-u_{i}\frac{1}{\omega }\intop _{0}^{\omega }a_{i}(t)u_{i}(t)dt +(1-h)[\overset{n}{\underset{j=1}{\sum }}f_{j}(u)\frac{1}{\omega }\intop _{0}^{\omega }K[b_{ij}(u_{j}(t))]dt +\overset{n}{\underset{j=1}{\sum }}g_{j}(u)\frac{1}{\omega }\intop _{0}^{\omega }K[c_{ij}(u_{j}(t))]dt\\ \displaystyle +\overset{n}{\underset{j=1}{\sum }}\frac{M}{\alpha }h_{j}(u)\frac{1}{\omega }\intop _{0}^{\omega }K[p_{ij}(u_{j}(t))]dt+\frac{1}{\omega }\intop _{0}^{\omega }I_{i}(t)dt]. \end{array} \end{aligned}$$

Which implies that

35 $$\begin{aligned} \displaystyle 0\notin \left( \phi \left( u,h\right) \right) _{i},\,i=1,2,\ldots ,n. \end{aligned}$$

If this is not true, then $0\in \left( \phi \left( u,h\right) \right) _{i},\,i=1,2,\ldots ,n,$, i.e.,

36 $$\begin{aligned} \displaystyle 0\in & -u_{i}\frac{1}{\omega }\intop _{0}^{\omega }a_{i}(t)u_{i}(t)dt+(1-h)[\overset{n}{\underset{j=1}{\sum }}f_{j}(u)\frac{1}{\omega }\intop _{0}^{\omega }K[b_{ij}(u_{i}(t))]dt +\overset{n}{\underset{j=1}{\sum }}g_{j}(u)\frac{1}{\omega }\intop _{0}^{\omega }K[c_{ij}(u_{i}(t))]dt\\ & \displaystyle \quad +\overset{n}{\underset{j=1}{\sum }}\frac{M}{\alpha }h_{j}(u)\frac{1}{\omega }\intop _{0}^{\omega }K[p_{ij}(u_{i}(t))]dt+\frac{1}{\omega }\intop _{0}^{\omega }I_{i}(t)dt]. \end{aligned}$$

Similarly, there exist $\gamma _{ij}(t)\in K[b_{ij}(u_{i})]$, $\eta _{ij}(t)\in K[c_{ij}(u_{i})]$ and $\nu _{ij}(t)\in K[p_{ij}(u_{i})]$, $i=1,2,\ldots ,n$ such that

37 $$\begin{aligned} 0= & {} (-u_{i})\frac{1}{\omega }\intop _{0}^{\omega }a_{i}(t)dt+(1-h)[\overset{n}{\underset{j=1}{\sum }}f_{j}(u)\frac{1}{\omega }\intop _{0}^{\omega }\gamma _{ij}(t)dt +\overset{n}{\underset{j=1}{\sum }}g_{j}(u)\frac{1}{\omega }\intop _{0}^{\omega }\eta _{ij}(t))dt+\overset{n}{\underset{j=1}{\sum }}\frac{M}{\alpha }h_{j}(u)\frac{1}{\omega }\intop _{0}^{\omega }\nu _{ij}(t))dt\nonumber \\{} & {} +\frac{1}{\omega }\intop _{0}^{\omega }I_{i}(t)dt],\,i=1,2,..,n, \end{aligned}$$

consequently, there exists some $$t^{**}\in [0,\omega ]$$ such that

38 $$\begin{aligned} \displaystyle 0 &=-u_{i}a_{i}(t^{**})+(1-h)[\overset{n}{\underset{j=1}{\sum }}\gamma _{ij}(t^{**})f_{j}(u)+\eta _{ij}(t^{**})g_{j}(u)\\ & \displaystyle \quad +\frac{M}{\alpha }\nu _{ij}(t^{**})h_{j}(u)+I_{i}(t^{**})]\,i=1,2,\ldots ,n. \end{aligned}$$

We derive from (38) that

$$\begin{aligned}\zeta _{i}^{*}&=|u_{i}|=\frac{1-h}{a_{i}(t^{**})}[\overset{n}{\underset{j=1}{\sum }}\gamma _{ij}(t^{**})f_{j}(u)+\eta _{ij}(t^{**})g_{j}(u)+\frac{M}{\alpha }\nu _{ij}(t^{**})h_{j}(u)+I_{i}(t^{**})]\\ &\le \frac{1}{a_{i}^{l}}[\overset{n}{\underset{j=1}{\sum }}(b_{ij}^{u}\left( F_{j}(|u_{j}|)+|f_{j}(0)|\right) +c_{ij}^{u}\left( G_{j}(|u_{j}|)+|g_{j}(0)|\right) +\frac{M}{\alpha }p_{ij}^{u}\left( H_{j}(|u_{j}|)+|h_{j}(0)|\right) )+I_{i}^{u}]\\ &\le \frac{1}{a_{i}^{l}}[\overset{n}{\underset{j=1}{\sum }}(b_{ij}^{u}\left( F_{j}(|u_{j}|)+|f_{j}(0)|\right) +\frac{c_{ij}^{u}\left( G_{j}(|u_{j}|)+|g_{j}(0)|\right) }{\sqrt{1-\tau _{ij}^{D}}}+\frac{M}{\alpha }p_{ij}^{u}\left( H_{j}(|u_{j}|)+|h_{j}(0)|\right) )+I_{i}^{u}]\\ &=\overset{n}{\underset{j=1}{\sum }}q_{ij}|u_{j}|+\theta _{i}=\overset{n}{\underset{j=1}{\sum }}q_{ij}\zeta _{j}^{*}+\theta _{i},\end{aligned}$$

which yields that $(I-S)\zeta ^{*}\le \theta$, which contradicts $(I-S)\zeta ^{*}>\theta$. Thus, (30) holds. Which implies that $(0,0,\ldots ,0)^{T}\notin \phi (u,h)$ for any $u=(u_{1},u_{2},\ldots ,u_{n})^{T}\partial \Delta \cap \mathbb {R}^{n},h\in [0,1]$. Thus, using the solution properties of the topological degree and the homotopy invariance, we have $deg\left\{ g_{0},\Delta \cap \mathbb {R}^{n},0\right\} =deg\left\{ \phi \left( u,0\right) ,\triangle \cap \mathbb {R}^{n},0\right\} =deg\left\{ \phi \left( u,1\right) ,\triangle \cap \mathbb {R}^{n},0\right\} =deg\left\{ \left( -\bar{a}_{1}u_{1},-\bar{a}_{2}u_{2},\ldots ,-\bar{a}_{n}u_{n}\right) ^{T},\Delta \cap \mathbb {R}^{n},0\right\}$

39 $$\begin{aligned} \begin{array}{l} \displaystyle =sign|\begin{array}{ccc} -\bar{a}_{1} &{} \ldots &{} 0\\ \vdots &{} \ddots &{} \vdots \\ \displaystyle 0 &{} \cdots &{} -\bar{a}_{1} \end{array}|=\left( -1\right) ^{n}\ne 0.\end{array} \end{aligned}$$

This means that $\triangle$ satisfies all the conditions in Lemma 2, then the system (1) possesses at least one $\omega -$periodic solution.

The proof is finished. $\square$

## Uniqueness and global exponential stability

Now, we will prove the uniqueness and global exponential stability of the $\omega$-periodic solution for the system (1). Mainly, when the system (1) is considered autonomous, we will find the sufficient conditions on the existence, uniqueness and global exponential stability of fixed point of the system.

### Definition 6

*(Stability)* We denote $x^{*}(t,\varphi )$ a periodic solution of the system (1). The periodic solution $x^{*}(t,\psi )$ is said to be globally exponentially stable if for any solution $x(t,\varphi )$ of the system (1), there are constants $M\ge 1$ and $\mu >0$ such that for any $\varphi \in C_{\tau }$

$$\left\| x(t,\varphi )-x^{*}(t,\psi )\right\| \le M\left\| \varphi -\psi \right\| _{C}e^{\mu t},\,t\ge 0.$$

Let us firstly introduce the following lemma.

### Lemma 3

^10^ If $f_{j}(\pm T_{j})=0$ , $g_{j}(\pm T_{j})=0$ and $h_{j}(\pm T_{j})=0$ $\left( j=1,\ldots ,n\right)$ then for every $x_{j},y_{j}\in \mathbb {R}$ we have

40 $$\begin{aligned} \displaystyle K[b_{ij}(x_{j}(t))]f_{j}(x_{j})-K[b_{ij}(y_{j}(t))]f_{j}(y_{j})\le b_{ij}^{u}F_{j}|x_{j}-y_{j}|, \end{aligned}$$

and

41 $$\begin{aligned} \displaystyle K[c_{ij}(x_{j}(t))]G_{j}(x_{j})-K[c_{ij}(y_{j}(t))]g_{j}(y_{j})\le c_{ij}^{u}G_{j}|x_{j}-y_{j}|, \end{aligned}$$

and

42 $$\begin{aligned} \displaystyle K[p_{ij}(x_{j}(t))]H_{j}(x_{j})-K[p_{ij}(y_{j}(t))]h_{j}(y_{j})\le p_{ij}^{u}H_{j}|x_{j}-y_{j}|, \end{aligned}$$

for $$i,j=1,2,\ldots ,n.$$

### Lemma 4

^43^ Let $x(t):[0,+\infty [\rightarrow \mathbb {R}^{n}$ an absolutely continuous on any compact interval of $[0,+\infty [$ and $V(x):\mathbb {R}^{n}\rightarrow \mathbb {R}$ is Clarke’s regular, then *x*(*t*) and $V(x):\mathbb {R}^{n}\rightarrow \mathbb {R}$ are differential for all $t\in [0,+\infty [$. We get

$$\frac{d}{dt}\nu (t)=\gamma (t)^{T}\dot{x}(t),\forall \gamma (t)\in \partial V(x(t)),$$

where $\partial V(x(t))$ is Clarke’s generalized gradient.

Next, we consider the assumption below.

**(H5)** $I-S$ is an $M-$matrix, *I* is the identity matrix of size $n,\,S=\left( s_{ij}\right) _{n\times n}$ and

$$s_{ij}=\frac{1}{a_{i}^{l}}\left( b_{ij}^{u}F_{j}+c_{ij}^{u}G_{j}+\frac{M}{\alpha }p_{ij}^{u}H_{j}\right) ,\,i,j=i,j=1,2,\ldots ,n.$$

### Theorem 2

Suppose that $f_{j}(\pm T_{j})=0,$ $g_{j}(\pm T_{j})=0$and $h_{j}(\pm T_{j})=0$ for $j=1,\ldots ,n$, and the assumption (H5) holds. If there exists periodic solution $x^{*}(t,\psi )$ for system (1), then $x^{*}(t,\psi )$ is a unique periodic solution of system (1) and is globally exponentially stable, and for any other solution $x(t,\varphi )$ of system (1), there exist constants $M,\,\mu >0$ such that

$$\begin{aligned} \left\| x_{i}(t,\varphi )-x_{i}^{*}(t,\psi )\right\| \le R\left\| \varphi -\psi )\right\| _{c}e^{-\mu t}, \end{aligned}$$

for any $$t>0.$$

### Proof

Consider $x(t)=(x_{1}(t),x_{2}(t),\ldots ,x_{n}(t))^{T}$ any solution of system (1) and $x^{*}(t)=(x_{1}^{*}(t),x_{2}^{*}(t),\ldots ,x_{n}^{*}(t))^{T}$ is an $\omega -$periodic solution of system (1). We get:

$$\begin{array}{l} \frac{dx_{i}(t)}{dt}\in -a_{i}x_{i}(t)+\overset{n}{\underset{j=1}{\sum }}K[b_{ij}(x_{j}(t))]f_{j}\left( x_{j}(t)\right) +\overset{n}{\underset{j=1}{\sum }}K[c_{ij}(x_{j}(t-\tau _{j}(t)))]g_{j}\left( x_{j}\left( t-\tau _{j}(t)\right) \right) \\ +\overset{n}{\underset{j=1}{\sum }}K[p_{ij}(x_{j}(t))]\overset{t}{\underset{-\infty }{\int }}h_{j}\left( x_{j}(s)\right) ds+J_{i}], \end{array}$$

$$\begin{array}{c} \frac{dx_{i}^{*}(t)}{dt}\in -a_{i}x_{i}^{*}(t)+\overset{n}{\underset{j=1}{\sum }}K[b_{ij}(x_{j}^{*}(t))]f_{j}\left( x_{j}^{*}(t)\right) +\overset{n}{\underset{j=1}{\sum }}K[c_{ij}(x_{j}^{*}(t-\tau _{j}(t)))]g_{j}\left( x_{j}\left( t-\tau _{j}(t)\right) \right) \\ +\overset{n}{\underset{j=1}{\sum }}K[p_{ij}(x_{j}^{*}(t))]\overset{t}{\underset{-\infty }{\int }}k_{ij}(t-s)h_{j}\left( x_{j}(s)\right) ds+J_{i}]. \end{array}$$

Assume that $y_{i}(t)=x_{i}(t)-x^*_{i}(t)$, then

43 $$\begin{aligned} \begin{array}{c} \displaystyle \frac{dy_{i}(t)}{dt}\in -a_{i}y_{i}(t)+\overset{n}{\underset{j=1}{\sum }}B_{ij}(y_{j}(t),x_{j}^{*}(t)) +\overset{n}{\underset{j=1}{\sum }}C_{ij}(y_{j}(t-\tau _{ij}(t),x_{j}^{*}(t-\tau _{ij}(t))) +\overset{n}{\underset{j=1}{\sum }}\frac{M}{\alpha }P_{ij}(y_{j}(t),x_{j}^{*}(t)), \end{array} \end{aligned}$$

where $B_{ij}(u,v)$, $C_{ij}(u,v)$ and $P_{ij}(u,v)$ are given as following

$$\begin{aligned} {\left\{ \begin{array}{ll} B_{ij}(u,v) &{} =K[b_{ij}(u+v)]f_{j}\left( u,v\right) -K[b_{ij}(v)]f_{j}\left( x_{j}(t)\right) \\ C_{ij}(u,v) &{} =K[c_{ij}(u+v)]g_{j}\left( u,v\right) -K[c_{ij}(v)]g_{j}\left( x_{j}(t)\right) \\ P_{ij}(u,v) &{} =K[p_{ij}(u+v)]h_{j}\left( u,v\right) -K[p_{ij}(v)]h_{j}\left( x_{j}(t)\right) \end{array}\right. }. \end{aligned}$$

Similarly, there exist $\gamma _{ij}(y_{j}(t))\in B_{ij}(y_{j}(t),x_{j}^{*}(t))$, $\eta _{ij}(y_{j}(t-\tau _{ij}(t)))\in C_{ij}(y_{j}(t-\tau _{ij}(t)),x_{j}^{*}(t-\tau _{ij}(t)))$ and $\nu _{ij}(y_{j}(t))\in P_{ij}(y_{j}(t),x_{j}^{*}(t))$ verify,

44 $$\begin{aligned} \begin{array}{c} \frac{dy_{i}(t)}{dt}\in -a_{i}y_{i}(t)+\overset{n}{\underset{j=1}{\sum }}\gamma _{ij}(y_{j}(t),y_{j}(t)) +\overset{n}{\underset{j=1}{\sum }}\eta _{ij}(y_{j}(t-\tau _{ij}(t),y_{j}(t-\tau _{ij}(t)))+\overset{n}{\underset{j=1}{\sum }}\frac{M}{\alpha }\nu _{ij}(y_{j}(t),y_{j}(t)) \end{array}, \end{aligned}$$

for every $$t\in [0,T),$$ $$i=1,2,\ldots ,n$$

Taking (44) and Lemma 3 into account, we obtain

$${\left\{ \begin{array}{ll} \gamma _{ij}(y_{j}(t)) &{} \le b_{ij}^{u}F_{j}|y_{j}(t)|\\ \eta _{ij}(y_{j}(t-\tau _{ij}(t))) &{} \le c_{ij}^{u}G_{j}|y_{j}(t-\tau _{ij}(t))|\\ \nu _{ij}(y_{j}(t)) &{} \le p_{ij}^{u}H_{j}|y_{j}(t)|\end{array}\right. }$$

Obviously, basing on (H5), the matrix $diag(a_{1}^{l},a_{1}^{l},\ldots ,a_{n}^{l})-(b_{ij}^{u}F_{j}+c_{ij}^{u}G_{j}+\frac{M}{\alpha }p_{ij}^{u}H_{j})_{n\times n}$ is also a nonsingular Mmatrix. In addition, there exists a positive $\beta _{i}(i=1,2,\ldots ,n)$ such that

$$\beta _{i}a_{i}^{l}-(b_{ij}^{u}F_{j}+c_{ij}^{u}G_{j}+\frac{M}{\alpha }p_{ij}^{u}H_{j})>0,\,i=1,2,\ldots ,n,$$

As a result, there exists a sufficiently small positive number $\mu$ such that

45 $$\begin{aligned} \beta _{i}\left( a_{i}^{l}-\mu \right) -\overset{n}{\underset{j=1}{\sum }}\beta _{j}(b_{ij}^{u}F_{j}+c_{ij}^{u}G_{j}e^{\mu \tau }+\frac{M}{\alpha }p_{ij}^{u}H_{j})>0,\,i=1,2,\ldots ,n. \end{aligned}$$

We consider the Lyapunov function:

$$\begin{aligned} V(t)=max\left\{ \frac{e^{\mu t}|y_{i}(t)|}{\beta _{j}},i=1,2,\ldots ,n.\right\} . \end{aligned}$$

*V*(*t*) is differential for all $t\ge 0$ because any solution *x*(*t*) of system (1) including the $\omega$-periodic solution $x^{*}(t)$ are absolutely continuous.

The function $|y_{i}(t)|$ is locally Lipschitz continuous in $y_{i}$ on $\mathbb {R}$. Hence, the Clarke’s generalized gradient of function $|y_{i}(t)|$ at $y_{i}(t)$ is

$$\partial (|y_{i}(t)|)=\bar{co}\left[ sign(y_{i}(t))\right] ={\left\{ \begin{array}{ll} -1 &{} if\,y_{i}(t)<0,\\ \left[ -1,1\right] &{} if\,y_{i}(t)=0,\\ -1 &{} if\,y_{i}(t)>0. \end{array}\right. }$$

For a given $t\ge 0$, there exists a $k\in \left\{ 1,\ldots ,n\right\}$ such that $V(t)=\frac{e^{\mu t}|y_{k}(t)|}{\beta _{k}},$ and let $v_{k}(t)=sign(y_{k}(t)$, if $y_{k}(t)\ne 0$, while $v_{k}(t)$ can be arbitrarily chosen in $[-1,1]$, if $y_{k}(t)=0$. From Lemma 4 and system (44), it follows for all $t\ge 0$ :

46 $$\begin{aligned} \dot{V}(t)=\mu V(t)+ V_{k}(t)\frac{e^{\mu t}\dot{y}_{k}(t)|}{\beta _{k}}\le -(a_{k}(t)-\mu )V(t)+\frac{e^{\mu t}}{\beta _{k}}\overset{n}{\underset{j=1}{\sum }}|\gamma _{ij}(y_{j}(t))| +\frac{e^{\mu t}}{\beta _{k}}\overset{n}{\underset{j=1}{\sum }}|\eta _{ij}(y_{j}(t-\tau _{ij}(t)))|+\frac{e^{\mu t}}{\beta _{k}}\overset{n}{\underset{j=1}{\sum }}\frac{M}{\alpha }|\nu _{ij}(y_{j}(t))|\\ \le -\left( a_{k}^{l}-\mu \right) V(t)+\frac{1}{\beta _{k}} \left[ \overset{n}{\underset{j=1}{\sum }}b_{ij}^{u}F_{j}\beta _{j}V(t)+\overset{n}{\underset{j=1}{\sum }}c_{ij}^{u}G_{j}\beta _{j}e^{\mu \tau _{ij}(t)}V(t-\tau _{ij}(t)) +\overset{n}{\underset{j=1}{\sum }}\frac{M}{\alpha }p_{ij}^{u}H_{j}\beta _{j}V(t)\right]\\ \le -\frac{1}{\beta _{k}}\left[ -\left( a_{k}^{l}-\mu \right) \beta _{k}-\overset{n}{\underset{j=1}{\sum }}\left( b_{ij}^{u}F_{j}+c_{ij}^{u}G_{j}e^{\mu \tau }+\frac{M}{\alpha }p_{ij}^{u}H_{j}\right) \beta _{j}\right] V(t)\le 0, \end{aligned}$$

when $V(t+s)\le V(t)$ for any $s\in \left[ -\tau ,0\right]$. Let $\bar{V}(t)=\underset{-\tau \le s\le 0}{sup}V(t+s),$ then we get

47 $$\begin{aligned} \frac{\bar{V}(t)}{dt}\le 0,\forall t \ge -\tau \end{aligned}$$

Therefore

48 $$\begin{aligned} \displaystyle |y_{j}(t)|\le \beta _{i}V(t)e^{-\mu t}\le \beta _{i}V(0)e^{-\mu t}, \end{aligned}$$

for all $i=1,\ldots ,n$. Thus, for any $t>0$,

$$\begin{aligned} \left\| y(t)\right\| \le y_{k}(t)(t)e^{-\mu t}\overset{n}{\underset{i=1}{\sum }}\beta _{i}/\beta _{k}. \end{aligned}$$

Moreover,

$$\begin{aligned} \left\| x(t)-x^{*}(t)\right\| \le R\left\| \varphi -\psi \right\| c^{e^{-\mu t}}, \end{aligned}$$

where $$R=\overset{n}{\underset{i=1}{\sum }}\beta _{i}/\beta _{min},\,x(t)=x(t,\varphi )$$ and $$x^{*}(t)=x^{*}(t,\psi )$$.

Hence, the $\omega$-periodic solution $x^{*}(t)$ of system (1) is globally exponentially stable. Then, the periodic solution $x^{*}(t)$ of system (1) is unique. The proof is complete. $\square$

### Theorem 3

Consider that $f_{j}(\pm T_{j})=0$, $g_{j}(\pm T_{j})=0$ and $h_{j}(\pm T_{j})=0$ $(j=1,\ldots ,n)$, and the assumption (H4) is satisfied. Then system (1) has a unique periodic solution $x^{*}(t,\psi )$, and it is globally exponentially stable.

Next, we demonstrate the existence and global exponential stability of the equilibrium point for autonomous neural network model (1).

Let $a_{i}^{l}=a_{i},\,b_{ij}^{u}=max\left\{ |\hat{b}_{ij}|,|\check{b}_{ij}|\right\} ,$ $c_{ij}^{u}=max\left\{ |\hat{c}_{ij}|,|\check{c}_{ij}|\right\}$ $p_{ij}^{u}=max\left\{ |\hat{p}_{ij}|,|\check{p}_{ij}|\right\}$ in the assumption (H4) and (H5) for system (1).

Firstly, for autonomous system (1), using Theorems 1 and 3 we can get the following result.

### Corollary 1

Consider that $f_{j}(\pm T_{j})=0$ and $g_{j}(\pm T_{j})=0$, $h_{j}(\pm T_{j})=0$ and $\tau _{ij}(t)\equiv \tau _{ij}$, where $\tau _{ij}\left( i,j=1,2,\ldots ,n\right)$ are all nonnegative constants. if (H5) is satisfied, then there exists a unique equilibrium point $x^{*}$ for system (1), which is globally exponentially stable.

### Proof

Clearly, system (1) is an $\omega$-periodic system, then, basing on Theorems 1 and 2, for any constant $\omega >0$ system (1) possesses a unique $\omega$-periodic solution $x^{*}(t)$ and it is globally exponentially stable.

Let $x^{*}(t)$ be unique for all $\omega >0$ , then we have $x^{*}(t+\omega )=x^{*}(t)$ for any constants $\omega >0$ and $t\ge 0$. Hence $x^{*}(t)\equiv x^{*}$ for all $t\ge 0$.

Thus $x^{*}=x^{*}(0)$ is an equilibrium point of system (1) and $x^{*}$ is unique and globally exponentially stable.

### Theorem 4

Consider that $f_{j}(\pm T_{j})=0$ and $g_{j}(\pm T_{j})=0\left( j=1,2,\ldots ,n\right)$. Since (H5) holds, there exists an uniqueness equilibrium point $x^{*}$ for system (1), which is globally exponentially stable.

From the assumption (H5), there exist positive constants $\beta _{i}\left( i=1,2,\ldots ,n\right)$ such that

$$\beta _{i}a_{i}^{l}-\overset{n}{\underset{j=1}{\sum }}\beta _{j}\left( b_{ij}^{u}F_{j}+c_{ij}^{u}G_{j}+\frac{M}{\alpha }p_{ij}^{u}H_{j}\right) >0,i=1,\ldots ,n.$$

After that, let a set-valued map $\Gamma (u)=\left( \Gamma _{1}(u),\Gamma _{1}(u),\ldots ,\Gamma _{n}(u)\right) ^{T}$, and

49 $$\begin{aligned} \begin{array}{c} \displaystyle \Gamma _{i}(u)=\beta _{i}[\overset{n}{\underset{j=1}{\sum }}K[b_{ij}(\frac{u_{j}}{\beta _{j}a_{i}})]f_{j}\left( \frac{u_{j}}{\beta _{j}a_{i}}\right) +\overset{n}{\underset{j=1}{\sum }}K[c_{ij}(\frac{u_{j}}{\beta _{j}a_{i}})]g_{j}\left( x_{j}\left( \frac{u_{j}}{\beta _{j}a_{i}}\right) \right) \\ +\overset{n}{\underset{j=1}{\sum }}K[p_{ij}(\frac{u_{j}}{\beta _{j}a_{i}})]\overset{t}{\underset{-\infty }{\int }}k_{ij}(t-s)h_{j}\left( x_{j}\left( \frac{u_{j}}{\beta _{j}a_{i}}\right) \right) ds+J_{i}] \end{array}, \end{aligned}$$

for $$i=1,2,\ldots ,n,$$ where $$u=\left( u_{1},\ldots ,u_{n}\right) ^{T}$$ .

Using Lemma 3, for any two vectors $u=(u_{1},\ldots ,u_{n})^{T}\in \mathbb {R}^{n}$ and $v=(v_{1},\ldots ,v_{n})^{T}\in \mathbb {R}^{n}$, we have

50 $$\begin{aligned} \displaystyle |\Gamma _{i}(u)-\Gamma _{i}(v)|&=\beta _{i}[\overset{n}{\underset{j=1}{\sum }}K[b_{ij}(\frac{u_{j}}{\beta _{j}a_{i}})]f_{j}\left( \frac{u_{j}}{\beta _{j}a_{i}}\right) -K[b_{ij}(\frac{u_{j}}{\beta _{j}a_{i}})]f_{j}\left( \frac{v_{j}}{\beta _{j}a_{i}}\right) +\overset{n}{\underset{j=1}{\sum }}K[c_{ij}(\frac{u_{j}}{\beta _{j}a_{i}})]g_{j}\left( x_{j}\left( \frac{u_{j}}{\beta _{j}a_{i}}\right) \right) \\ & \displaystyle \quad -\overset{n}{\underset{j=1}{\sum }}K[c_{ij}(\frac{u_{j}}{\beta _{j}a_{i}})]g_{j}\left( x_{j}\left( \frac{v_{j}}{\beta _{j}a_{i}}\right) \right) +\overset{n}{\underset{j=1}{\sum }}\frac{M}{\alpha }K[p_{ij}(\frac{u_{j}}{\beta _{j}a_{i}}]h_{j}\left( \frac{u_{j}}{\beta _{j}a_{i}}\right) ds-\overset{n}{\underset{j=1}{\sum }}\frac{M}{\alpha }K[p_{ij}(\frac{u_{j}}{\beta _{j}a_{i}})]h_{j}\left( \frac{v_{j}}{\beta _{j}a_{i}}\right) ], \end{aligned}$$

for $$i=1,2,\ldots ,n,$$ then,

51 $$\begin{aligned} \displaystyle \left\| \Gamma _{i}(u)-\Gamma _{i}(v)\right\| \le \sigma \left\| u-v\right\| , \end{aligned}$$

where

52 $$\begin{aligned} \displaystyle \sigma =\underset{1\le i\le n}{max}\left\{ \frac{1}{\beta _{i}a_{i}^{l}}\overset{n}{\underset{j=1}{\sum }}\beta _{j}\left( b_{ij}^{u}F_{j}+c_{ij}^{u}G_{j}+\frac{M}{\alpha }p_{ij}^{u}H_{j}\right) \right\} , \end{aligned}$$

and $0<\sigma <1$. Thus, the map $\Gamma :\mathbb {R}^{n}\rightarrow \mathbb {R}^{n}$ is a contraction mapping on $\mathbb {R}^{n}$. It follows that, there is a unique fixed point $u^{*}\in \mathbb {R}^{n}$ such that $u^{*}\in \Gamma (u^{*})$, i.e.,

$$u_{i}^{*}\in \beta _{i}[\overset{n}{\underset{j=1}{\sum }}K[b_{ij}(\frac{u_{j}^{*}}{\beta _{j}a_{i}})]f_{j}\left( \frac{u_{j}^{*}}{\beta _{j}a_{i}}\right) +\overset{n}{\underset{j=1}{\sum }}K[c_{ij}(\frac{u_{j}^{*}}{\beta _{j}a_{i}})]g_{j}\left( \frac{u_{j}^{*}}{\beta _{j}a_{i}}\right) +\overset{n}{\underset{j=1}{\sum }}\frac{M}{\alpha }K[p_{ij}(\frac{u_{j}^{*}}{\beta _{j}a_{i}})]h_{j}\left( \frac{u_{j}^{*}}{\beta _{j}a_{i}}\right) +J_{i}]$$

for $i=1,\ldots ,n$. Let $x_{j}^{*}=\frac{u_{j}^{*}}{\beta _{j}a_{i}}$ for $i=1,\ldots ,n$, then

$$u_{i}^{*}\in -a_{i}x_{i}^{*}+\overset{n}{\underset{j=1}{\sum }}K[b_{ij}(x_{j}^{*})]f_{j}\left( x_{j}^{*}\right) +\overset{n}{\underset{j=1}{\sum }}K[c_{ij} (x_{j}^{*})]g_{j}\left( x_{j}^{*}\right) +\overset{n}{\underset{j=1}{\sum }}\frac{M}{\alpha }K[p_{ij}(x_{j}^{*})]h_{j}\left( x_{j}^{*}\right) +J_{i}.$$

where $i=1,\ldots ,n$, and *u* is unique, we obtain that system (1) has a unique equilibrium $x^{*}$.

Thus, following the proof of Theorem 1, we prove easily that equilibrium $x^{*}$ of system (1) is globally exponentially stable.

## Finite-time periodic synchronization

In this section, we will examine the finite-time synchronization problem of delayed memristive neural networks.

For this purpose, we consider the delayed memristive neural network model (1) as the drive system, and a controlled response system is modeled by the following functional differential equation:

53 $$\begin{aligned} \dot{y}_{i}(t)= & {} -a_{i}(t)y_{i}(t)+\overset{n}{\underset{j=1}{\sum }}[b_{ij}(t)f_{j}(y_{j}(t)) +c_{ij}(t)g_{j}(y_{j}(t-\tau _{ij}(t)))\nonumber \\{} & {} +p_{ij}(t)\overset{t}{\underset{-\infty }{\int }}k_{ij}(t-s)h_{j}(y_{j}(s))ds+J_{i}(t)]+v_{i}(t) \end{aligned}$$

where $y_{i}(t)$ is the controller to be designed.

### Definition 7

The memristive neural network (1) is said to be completely synchronized onto (53) in finite time if by designing a suitable controller $v_{i}(t)$ to system (53), there exists a constant $t_{1}$>0 ($t_{1}$ depends on the initial value), satisfying

$$\begin{aligned} \begin{array}{c} \displaystyle \underset{t\rightarrow t_{1}}{lim}\left\| y_{i}(t)-x_{i}(t)\right\| =0;\\ \displaystyle \left\| y_{i}(t)-x_{i}(t)\right\| \equiv 0,\,for\,i=1,2,..n,\,t>t_{1} \end{array} \end{aligned}$$

We take $e_{i}(t)=x_{i}(t)-y_{i}(t)$ the error term. Then, one can obtain the following result.

### Theorem 5

We consider that then system (1) exists at least one w-periodic solution. If there exists a positive definite matrix *S* satisfying

$$\begin{array}{c} Z_{1}=\left( \begin{array}{cc} -A+\frac{1}{2}S+\underline{B}F+\frac{M}{\alpha }\underline{P}H &{} \frac{1}{2}\underline{C}G\\ * &{} -\frac{1}{2}S \end{array}\right) <0.\end{array}$$

$$\begin{array}{c} Z_{2}=\left( \begin{array}{cc} -A+\frac{1}{2}S+\overline{B}F+\frac{M}{\alpha }\underline{P}H &{} \frac{1}{2}\overline{C}G\\ * &{} -\frac{1}{2}S \end{array}\right) <0,\end{array}$$

where $F=diag(F_{1},F_{2},\ldots F_{n})$, $G=diag(G_{1},G_{2},\ldots G_{n})$, $H=diag(H_{1},H_{2},\ldots H_{n})$, $\underline{B}=diag(\underline{b}_{1},\underline{b}_{2},\ldots \underline{b}_{n})$, $\overline{B}=diag(\overline{b}_{1},\overline{b}_{2},\ldots \overline{b}_{n})$, $\underline{C}=diag(\underline{c}_{1},\underline{c}_{2},\ldots \underline{c}_{n})$, $\overline{C}=diag(\overline{c}_{1},\overline{c}_{2},\ldots \overline{c}_{n})$, then system (53) can synchronize onto system (1) in a finite time $t_{1}=\frac{\sqrt{2}}{k}V^{\frac{1}{2}}\left( 0\right)$ and to adapt to changes in the process that occur with time, we define the adaptive controller

54 $$\begin{aligned} v_{i}(t)=-o_{i}(t)e_{i}(t), \end{aligned}$$

and adaptive updated law, where

$$\begin{array}{c} \dot{o}_{i}(t)=\varepsilon _{i}\left( e_{i}^{2}\left( t\right) -l_{i}\frac{e_{i}\left( t\right) }{o_{i}(t)}sign(e_{i}\left( t\right) )-k\frac{e_{i}\left( t\right) }{o_{i}(t)}sign(e_{i}\left( t\right) )-\frac{k}{\sqrt{\varepsilon _{i}}}sign(o_{i}\left( t\right) )\right) -k\sqrt{\lambda _{max}(P)}\left( \int _{t-\tau }^{0}e_{i}^{2}(s)ds\right) ^{\frac{1}{2}}), \end{array}$$

and

$$\begin{array}{c} V(0)=\frac{1}{2}e^{T}(0)e(0)+\frac{1}{2}\int _{-\tau }^{0}e^{T}(s)Se(s)ds+\frac{1}{2}\overset{n}{\underset{i=1}{\sum }}\frac{1}{\varepsilon _{i}}o_{i}^{2}(0).\end{array}$$

$\varepsilon _{i}>0$ is a constant, $k>0$ is a tunable constant,

$\iota _{i}>0,i=1,2,\ldots ,n$, are the control parameters to be determined and satisfies:

$$\begin{aligned} \begin{array}{c} \iota _{i}\ge |A|T_{i}+\overset{n}{\underset{j=1}{\sum }}F_{j}|\overline{b}_{ij}-\underline{b}_{ij}|T_{i}+\overset{n}{\underset{j=1}{\sum }}{\sum }|\overline{b}_{ij}-\underline{b}_{ij}|G_{j} \overset{n}{\underset{j=1}{\sum }}\frac{M}{\alpha }|\overline{p}_{ij}-\underline{p}_{ij}|H_{j} \end{array}. \end{aligned}$$

### Proof

Set $\Lambda =diag\left\{ o_{1}(t),o_{2}(t),\ldots o_{n}(t)\right\}$. Consider the following Lyapunov functional:

55 $$\begin{aligned} \begin{array}{c} V(t)=\frac{1}{2}e^{T}(t)e(t)+\frac{1}{2}\int _{t -\tau }^{0}e^{T}(s)Se(s)ds+\frac{1}{2} \overset{n}{\underset{i=1}{\sum }}\frac{1}{\varepsilon _{i}}o_{i}^{2}(t).\end{array} \end{aligned}$$

The master model (1) and the slave model (53) are state-dependent switching systems, hence, we can divide the error system into the following four cases at time *t*.

*Case 1* If $|x_{i}(t)|>T_{i}$, $|y_{i}(t)|\ge T_{i,}$ at time *t*, then the master system (1) and the slave system (53) decrease respectively, to the following models:

56 $$\begin{aligned} {\displaystyle \dot{x}_{i}(t)=-a_{i}(t)x_{i}(t)+\overset{n}{\underset{j=1}{\sum }}\underline{b}_{ij}(t)f_{j}(x_{j}(t))} {\displaystyle +\underline{c}_{ij}(t)g_{j}(x_{j}(t-\tau _{ij}(t)))+\underline{p}_{ij}(t)\overset{t}{\underset{-\infty }{\int }}k_{ij}(t-s)h_{j}(x_{j}(s))ds}+J_{i}(t), \end{aligned}$$

and

57 $$\begin{aligned} {\displaystyle \dot{y}_{i}(t)=-a_{i}(t)y_{i}(t)+\overset{n}{\underset{j=1}{\sum }}\underline{b}_{ij}(t)f_{j}(y_{j}(t))+\underline{c}_{ij}(t)g_{j}(y_{j}(t-\tau _{ij}(t)))} {\displaystyle +\underline{p}_{ij}(t)\overset{t}{\underset{-\infty }{\int }}k_{ij}(t-s)h_{j}(y_{j}(s))ds}+u_{i}(t)+J_{i}(t). \end{aligned}$$

Correspondingly, the error system can be written as

58 $$\begin{aligned} \dot{e}_{i}(t)=-a_{i}(t)e_{i}(t)+\overset{n}{\underset{j=1}{\sum }}\underline{b}_{ij}(t)f_{j}(e_{j}(t))+\underline{c}_{ij}(t)g_{j}(e_{j}(t-\tau _{ij}(t)))+\underline{p}_{ij}(t)\overset{t}{\underset{-\infty }{\int }}k_{ij}(t-s)h_{j}(e_{j}(s))ds+u_{i}(t). \end{aligned}$$

Let us denote $f_{j}(e_{j}(t))=f_{j}(x_{j}(t))-f_{j}(y_{j}(t))$; $g_{j}(e_{j}(t-\tau ))=g_{j}(x_{j}(t-\tau ))-g_{j}(y_{j}(t-\tau ))$ and $h_{j}(e_{j}(t))=h_{j}(x_{j}(t))-h_{j}(y_{j}(t))$. Under assumption (H2), evaluating the derivation of *V*(*t*) along the trajectory of error system gives

59 $$\begin{aligned} \dot{V}(t){} & {} = e^{T}(t)\left( -Ae(t)+\underline{B}f(e(t))+\underline{C}ge(t-\tau )+\frac{M}{\alpha }\underline{P}h(e(t))+u(t)\right) +\frac{1}{2}e^{T}(t)Se(t)\nonumber \\{} & {} \quad -\frac{1}{2}e^{T}(t-\tau )Se(t-\tau )\\{} & {} \quad +\frac{1}{2}\overset{n}{\underset{i=1}{\sum }}o_{i}(t)(e_{i}^{2}(t)-l_{i}\frac{e_{i}(t)}{o_{i}(t)}sign(e_{i}(t)) -k\frac{e_{i}(t)}{o_{i}(t)}sign(e_{i}(t))\nonumber \\{} & {} \quad -\frac{k}{\sqrt{\varepsilon _{i}}}sign(o_{i}(t))-k\sqrt{\lambda _{max}(S)}\left( \intop _{t-\tau }^{t}e_{i}^{2}(s)ds\right) ^{\frac{1}{2}}\bigg)\nonumber \\{} & {} \le -e^{T}(t)Ae(t)+e^{T}\underline{B}Fe(t)+e^{T}\underline{C}Ge(t-\tau )+e^{T}\frac{M}{\alpha }\underline{P}He(t)-e^{T}(t)\Lambda e(t)\nonumber \\{} & {} \quad +\frac{1}{2}e^{T}(t)Se(t)-\frac{1}{2}e^{T}(t-\tau )Se(t-\tau )\nonumber \\{} & {} \quad +e^{T}(t)\Lambda e(t)-\overset{n}{\underset{i=1}{\sum }}\iota _{i}|e_{i}(t)|-k\overset{n}{\underset{i=1}{\sum }}|e_{i}(t)|-k\overset{n}{\underset{i=1}{\sum }}\frac{1}{\sqrt{\varepsilon _{i}}}|o_{i}(t)|-k\left( \intop _{t-\tau }^{t}e^{T}(s)Se(s)ds\right) ^{\frac{1}{2}}\nonumber \\{} & {} \le \left( e^{T}(t),e^{T}(t-\tau )\right) Z_{1}\left( e^{T}(t),eT(t-\tau )\right) ^{T}-\overset{n}{\underset{i=1}{\sum }}\iota _{i}|e_{i}(t)-k\overset{n}{\underset{i=1}{\sum }}|e_{i}(t)||\nonumber \\{} & {} \quad -k\overset{n}{\underset{i=1}{\sum }}\frac{1}{\sqrt{\varepsilon _{i}}}|o_{i}(t)|-k\left( \intop _{t-\tau }^{t}e^{T}(s)Se(s)ds\right) ^{\frac{1}{2}}.\nonumber \end{aligned}$$

Using previous results, we obtain

$$\begin{array}{c} \dot{V}(t)\le -k\left( \overset{n}{\underset{i=1}{\sum }}|e_{i}(t)|^{2}\right) ^{\frac{1}{2}}-k\left( \intop _{t-\tau }^{t}e^{T}(s)Se(s)ds\right) ^{\frac{1}{2}}-k\overset{n}{\underset{i=1}{\sum }}\frac{1}{\sqrt{\varepsilon _{i}}}|o_{i}(t)|. \end{array}$$

By Lemma 1, one has

$$\begin{array}{c} \dot{V}(t)\le \begin{array}{c} -\sqrt{2}k\left[ \frac{1}{2}e^{T}(t)e(t)+\frac{1}{2}\int _{t-\tau }^{t}e^{T}(s)Se(s)ds+\frac{1}{2}\overset{n}{\underset{i=1}{\sum }}\frac{1}{\varepsilon _{i}}o_{i}^{2}(t)\right] ^{\frac{1}{2}}\end{array} =-\sqrt{2}kV^{\frac{1}{2}}(t). \end{array}$$

*Case 2* Let $|x_{i}(t)|>T_{i}$ ,$|y_{i}(t)|>T_{i}$ at time *t*, then the master system (1) and the slave system (53) decrease to the following systems:

60 $$\begin{aligned} \begin{array}{c} \begin{array}{l} {\displaystyle \dot{x}_{i}(t)=-a_{i}(t)x_{i}(t)+\overset{n}{\underset{j=1}{\sum }}\overline{b}_{ij}(t)f_{j}(x_{j}(t))} {\displaystyle +\overset{n}{\underset{j=1}{\sum }}\overline{c}_{ij}(t)g_{j}(x_{j}(t-\tau _{ij}(t)))+\overset{n}{\underset{j=1}{\sum }}\overline{p}_{ij}(t)\overset{t}{\underset{-\infty }{\int }}k_{ij}(t-s)h_{j}(x_{j}(s))ds}+J_{i}(t) \end{array},\end{array} \end{aligned}$$

and

61 $$\begin{aligned} \dot{y}_{i}(t)=-a_{i}(t)y_{i}(t)+\overset{n}{\underset{j=1}{\sum }}\overline{b}_{ij}(t)f_{j}(y_{j}(t))+\overline{c}_{ij}(t)g_{j}(y_{j}(t-\tau _{ij}(t))) +\overline{p}_{ij}(t)\overset{t}{\underset{-\infty }{\int }}k_{ij}(t-s)h_{j}(y_{j}(s))ds+J_{i}(t)+u_{i}(t). \end{aligned}$$

Hence, we obtain the following error system

62 $$\begin{aligned} {\displaystyle \dot{e}_{i}(t)=-a_{i}(t)e_{i}(t)+\overset{n}{\underset{j=1}{\sum }}\overline{b}_{ij}(t)f_{j}(e_{j}(t))+\overline{c}_{ij}(t)g_{j}(e_{j}(t-\tau _{ij}(t))) +\overline{p}_{ij}(t)\overset{t}{\underset{-\infty }{\int }}k_{ij}(t-s)h_{j}(e_{j}(s))ds+w_{i}(t)}. \end{aligned}$$

Similarly, we write

63 $$\begin{aligned} \dot{V}(t)\le & {} \left( e^{T}(t),e^{T}(t-\tau )\right) Z_{2}\left( e^{T}(t),e(t-\tau )\right) ^{T}-\overset{n}{\underset{i=1}{\sum }}l_{i}|e_{i}(t)-k\overset{n}{\underset{i=1}{\sum }}|e_{i}(t)||\nonumber \\{} & {} -k\overset{n}{\underset{i=1}{\sum }}\frac{1}{\sqrt{\varepsilon _{i}}}|o_{i}(t)|-k\left( \intop _{t-\tau }^{t}e^{T}(s)Se(s)ds\right) ^{\frac{1}{2}}. \end{aligned}$$

According to Lemmas 1, it follows

$$\begin{array}{c} \dot{V}(t)\le \begin{array}{c} -\sqrt{2}k\left[ \frac{1}{2}e^{T}(t)e(t)+\frac{1}{2}\int _{t-\tau }^{t}e^{T}(s)Se(s)ds+\frac{1}{2}\overset{n}{\underset{i=1}{\sum }}\frac{1}{\varepsilon _{i}}o_{i}^{2}(t)\right] ^{\frac{1}{2}}\end{array}\\ =-\sqrt{2}kV^{\frac{1}{2}}(t). \end{array}$$

*Case 3* If $|x_{i}(t)|>Ti$ , $|y_{i}(t)|\le T_{i}$ at time t, then the master system (1) and the slave system (53) reduce to (60) and (61). Correspondingly, the error system can be written as

64 $$\begin{aligned} &{\displaystyle \dot{e}_{i}(t) =-a_{i}(t)e_{i}(t)+\overset{n}{\underset{j=1}{\sum }}\overline{b}_{ij}(t)f_{j}(e_{j}(t))+\overset{n}{\underset{j=1}{\sum }}\overline{c}_{ij}(t)g_{j}(e_{j}(t-\tau _{ij}(t)))}\\& \quad {\displaystyle +\overset{n}{\underset{j=1}{\sum }}\overline{p}_{ij}(t)\overset{t}{\underset{-\infty }{\int }}k_{ij}(t-s)h_{j}(e_{j}(s))ds}+\left( \overline{a}_{i}(t)-\underline{a}_{i}(t)\right) y_{i}(t)\\ & \quad +\overset{n}{\underset{j=1}{\sum }}\left( \underline{b}_{ij}(t)-\overline{b}_{ij}(t)\right) f_{j}(y_{j}(t))+\overset{n}{\underset{j=1}{\sum }}\left( \underline{c}_{ij}(t)-\overline{c}_{ij}(t)\right) g_{j}(y_{j}(t-\tau _{ij}(t)))\\ & \quad {\displaystyle +\overset{n}{\underset{j=1}{\sum }}\left( \underline{p}_{ij}(t)-\overline{p}_{ij}(t)\right) \overset{t}{\underset{-\infty }{\int }}k_{ij}(t-s)h_{j}(y_{j}(s))ds+u_{i}(t).} \end{aligned}$$

evaluating the derivation of V(t) along the trajectory of (68), we have

$$\begin{aligned} \dot{V}_{i}(t){} & {} = \overset{n}{\underset{i=1}{\sum }}e_{i}(t)\bigg[-a_{i}(t)e_{i}(t)+\overset{n}{\underset{j=1}{\sum }}\overline{b}_{ij}(t)f_{j}(e_{j}(t))+\overset{n}{\underset{j=1}{\sum }}\overline{c}_{ij}(t)g_{j}(e_{j}(t-\tau _{ij}(t)))\\{} & {} \quad +\overset{n}{\underset{j=1}{\sum }}\overline{p}_{ij}(t)\overset{t}{\underset{-\infty }{\int }}k_{ij}(t-s)h_{j}(e_{j}(s))ds+|\underline{a}_{i}-\overline{a}_{i}||y_{i}(t)|\\{} & {} \quad +\overset{n}{\underset{j=1}{\sum }}|\underline{b}_{ij}-\overline{b}_{ij}||f_{j}(y_{j}(t))|+\overset{n}{\underset{j=1}{\sum }}|\underline{c}_{ij}-\overline{c}_{ij}||g_{j}(y_{j}(t-\tau _{ij}(t)))|+\overset{n}{\underset{j=1}{\sum }}|\underline{p}_{ij}-\overline{p}_{ij}|\overset{t}{\underset{-\infty }{\int }}k_{ij}(t-s)|h_{j}(y_{j}(s))|ds\\{} & {} \quad +u_{i (t)}] +\frac{1}{2}e^{T}(t)Se(t) -\frac{1}{2}e^{T}(t-\tau )Se(t-\tau )+\overset{n}{\underset{i=1}{\sum }}o_{i}^{2}(t)-l_{i}\frac{e_{i}(t)}{o_{i}(t)}sign(e_{i}(t)) -k\frac{e_{i}(t)}{o_{i}(t)}sign(e_{i}(t))\\{} & {} \quad -\frac{k}{\sqrt{\varepsilon _{i}}}sign(o_{i}(t)) -k\sqrt{\lambda _{max}(S)}\left( \int _{t-\tau }^{0}e_{i}^{2}(s)ds\right) ^{\frac{1}{2}}\bigg]\\{} & {} \le -e^{T}(t)Ae(t)+e^{T}\overline{B}Fe(t)+e^{T}\overline{C}Ge(t-\tau )+\frac{M}{\alpha }\overline{P}He(t)-e^{T}(t)\Lambda e(t) +\frac{1}{2}e^{T}(t)Se(t)\\{} & {} \quad -\frac{1}{2}e^{T}(t-\tau )Se(t-\tau )+e^{T}(t)\Lambda e(t)\\{} & {} \quad -\overset{n}{\underset{i=1}{\sum }}|e_{i}(t)|-k\overset{n}{\underset{i=1}{\sum }}|e_{i}(t)|-k\overset{n}{\underset{i=1}{\sum }}\frac{1}{\sqrt{\varepsilon _{i}}}|o_{i}(t)|-k\left( \intop _{t-\tau }^{t}e^{T}(s)Se(s)ds\right) ^{\frac{1}{2}} +\overset{n}{\underset{j=1}{\sum }}[|a_{i}|T_{i}\\{} & {} \quad +\overset{n}{\underset{j=1}{\sum }}F_{j}|\underline{b}_{ij}-\overline{b}_{ij}|T_{i}+\overset{n}{\underset{j=1}{\sum }}|\underline{c}_{ij}-\overline{c}_{ij}|G_{j}-l_{i}]|e_{i}(t)|\\{} & {} \quad +\overset{n}{\underset{j=1}{\sum }}\frac{M}{\alpha }H_{j}|\underline{p}_{ij}-\overline{p}_{ij}|\\\le & {} \left( e^{T}(t),e^{T}(t-\tau )\right) Z_{2}\left( e^{T}(t),e(t-\tau )\right) ^{T}-k\overset{n}{\underset{i=1}{\sum }}|e_{i}(t)|-k\overset{n}{\underset{i=1}{\sum }}\frac{1}{\sqrt{\varepsilon _{i}}}|o_{i}(t)|\\{} & {} \quad -k\left( \intop _{t-\tau }^{t}e^{T}(s)Se(s)ds\right) ^{\frac{1}{2}}+\overset{n}{\underset{j=1}{\sum }}[|a_{i}|T_{i}+\overset{n}{\underset{j=1}{\sum }}F_{j}|\overline{b}_{i}-\underline{b}_{ij}|T_{i}\\{} & {} \quad +\overset{n}{\underset{j=1}{\sum }}|\overline{c}_{ij}-\underline{c}_{ij}|G_{j} +\overset{n}{\underset{j=1}{\sum }}\frac{M}{\alpha }H_{j}|\underline{p}_{ij}-\overline{p}_{ij}|T_{i}-l_{i}]|e_{i}(t)|. \end{aligned}$$

In consideration of the definition $l_{i}$ and $Z_{2}$, one has $$\begin{array}{c} \dot{V}_{i}(t)\le -\sqrt{2}kV^{\frac{1}{2}}(t).\end{array}$$

*Case 4.* Let $|x_{i}(t) |\le T_{i}$ , $|y_{i}(t)|>T_{i}$ at time t, then the master system (1) and the slave system (53) reduce to (60) and (62). Then, we obtain the following error system:

65 $$\begin{aligned} \dot{e}_{i}(t)= & {} -a_{i}(t)e_{i}(t)+\overset{n}{\underset{j=1}{\sum }}\overline{b}_{ij}(t)f_{j}(e_{j}(t))+\overset{n}{\underset{j=1}{\sum }}\overline{c}_{ij}(t)g_{j}(e_{j}(t-\tau _{ij}(t)))\nonumber \\{} & {} +\overset{n}{\underset{j=1}{\sum }}\overline{p}_{ij}(t)\overset{t}{\underset{-\infty }{\int }}k_{ij}(t-s)h_{j}(e_{j}(s))ds+a_{i}(t)x_{i}(t) \nonumber \\{} & {} +\overset{n}{\underset{j=1}{\sum }}\left( \overline{b}_{i}(t)-\underline{b}_{ij}(t)\right) f_{j}(x_{j}(t))+\overset{n}{\underset{j=1}{\sum }}\left( \overline{c}_{ij}(t)-\underline{c}_{ij}(t)\right) g_{j}(x_{j}(t-\tau _{ij}(t)))\nonumber \\{} & {} +\overset{n}{\underset{j=1}{\sum }}\left( \overline{p}_{ij}(t)-\underline{p}_{ij}(t)\right) \overset{t}{\underset{-\infty }{\int }}k_{ij}(t-s)h_{j}\left( x_{j}(s)\right) ds+u_{i}(t). \end{aligned}$$

Consider $|x_{i}(t)|\le T_{i}$ , we obtain

$$\begin{aligned} \dot{V}_{i}(t)\le & {} \left( e^{T}(t),e^{T}(t-\tau )\right) Z_{2}\left( e^{T}(t),e(t- \tau )\right) ^{T}-k\overset{n}{\underset{i=1}{\sum }}|e_{i}(t)|-k\overset{n}{\underset{i=1}{\sum }}\frac{1}{\sqrt{\varepsilon _{i}}}|o_{i}(t)|\\{} & {} -k\left( \intop _{t-\tau }^{t}e^{T}(s)Se(s)ds\right) ^{\frac{1}{2}}+\overset{n}{\underset{j=1}{\sum }}[|a_{i}|T_{i}\\{} & {} +\overset{n}{\underset{j=1}{\sum }}F_{j}|\underline{b}_{i}-\overline{b}_{ij}|T_{i}+\overset{n}{\underset{j=1}{\sum }}|\underline{c}_{ij}-\overline{c}_{ij}|G_{j}+\overset{n}{\underset{j=1}{\sum }}|\underline{c}_{ij}-\overline{c}_{ij}|\frac{M}{\alpha }H_{j}-l_{i}|] e_{i}(t)\le -\sqrt{2}kV^{\frac{1}{2}}(t)|\end{aligned}$$

Or $V(t)=0$ for $t\ge t_{1}$ with $t_{1}=\frac{\sqrt{2}}{k}V^{\frac{1}{2}}(0)$ , hence $e_{i}(t)=0$ for $t\ge t_{1}$, $i=1,2,\ldots ,n$. According to definition 5, the salve system (53) is finite-timely synchronized onto the master system (1) under the designed controller (54). This completes the proof. $\square$

## Numerical example

In this section, numerical example is given to show the effectiveness of our results. We consider the two-dimensional mermristor-based recurrent neural networks described by the following system:

$$\begin{aligned} {\displaystyle \dot{x}_{i}(t)=-a_{i}x_{i}(t)+\overset{2}{\underset{j=1}{\sum }}(b_{ij}f_{j}(x_{j}(t)) +c_{ij}g_{j}(x_{j}(t-\tau _{ij}(t)))+p_{ij}\overset{t}{\underset{-\infty }{\int }}k_{ij}(t-s)h_{j}(x_{j}(s))ds+J_{i}(t)}, \end{aligned}$$

where $i=1,22$, $a_{1}=[3\,4]$, $\tau _{ij}(t)=\frac{1}{5}cos(t)$ and for all $x\in \mathbb {R}$

$$\begin{aligned} f_{j}(x)= & {} g_{j}(x)=h_{j}(x)=\phi _{j}(x)=\frac{|x+1|-|x-1|}{2}\\ b_{11}(x_{1}(t))= & {} {\left\{ \begin{array}{ll} -0.1, &{} |x_{1}(t)|<1\\ 1, &{} |x_{1}(t)|>1 \end{array}\right. }, b_{12}(x_{1}(t))={\left\{ \begin{array}{ll} cos(t), &{} |x_{1}(t)|<1\\ -0.5, &{} |x_{1}(t)|>1 \end{array}\right. }\\ b_{21}(x_{2}(t))= & {} {\left\{ \begin{array}{ll} -0.5*sin(t), &{} |x_{2}(t)|<1\\ 1, &{} |x_{2}(t)|>1 \end{array}\right. }, b_{22}(x_{2}(t))= {\left\{ \begin{array}{ll} 0.1*cos(t),\, &{} |x_{2}(t)|<1\\ -1, &{} |x_{2}(t)|>1 \end{array}\right. }\\ c_{11}(x_{1}(t))= & {} {\left\{ \begin{array}{ll} 0.5, &{} |x_{1}(t)|<1\\ 1, &{} |x_{1}(t)|>1 \end{array}\right. }, c_{12}(x_{1}(t))={\left\{ \begin{array}{ll} 2sin(t), &{} |x_{1}(t)|<1\\ -0.3*cos(-t), &{} |x_{1}(t)|>1 \end{array}\right. }\\ c_{21}(x_{2}(t))= & {} {\left\{ \begin{array}{ll} 0.2*sin(t), &{} |x_{2}(t)|<1\\ sin(t), &{} |x_{2}(t)|>1 \end{array}\right. }, c_{22}(x_{2}(t)={\left\{ \begin{array}{ll} 0.1*cos(t), &{} |x_{2}(t)|<1\\ -1, &{} |x_{2}(t)|>1 \end{array}\right. }\\ p_{11}(x_{1}(t))= & {} {\left\{ \begin{array}{ll} 0.5, &{} |x_{1}(t)|<1\\ -0.5, &{} |x_{1}(t)|>1 \end{array}\right. }, p_{12}(x_{1}(t))]={\left\{ \begin{array}{ll} 2sin(t), &{} |x_{1}(t)|<1\\ 1.5, &{} |x_{1}(t)|>1 \end{array}\right. }\\ p_{21}(x_{2}(t))= & {} {\left\{ \begin{array}{ll} -0.2*sin(t), &{} |x_{2}(t)|<1\\ 1, &{} |x_{2}(t)|>1 \end{array}\right. }, p_{22}(x_{2}(t))]={\left\{ \begin{array}{ll} 0.5*cos(t), &{} |x_{2}(t)|<1\\ 1, &{} |x_{2}(t)|>1 \end{array}\right. }\\ M= & {} 0.4, \alpha =5.\\ J= & {} [0.1*sin(t);\ 0.2*cos(t)]; \end{aligned}$$

**Figure 2:**
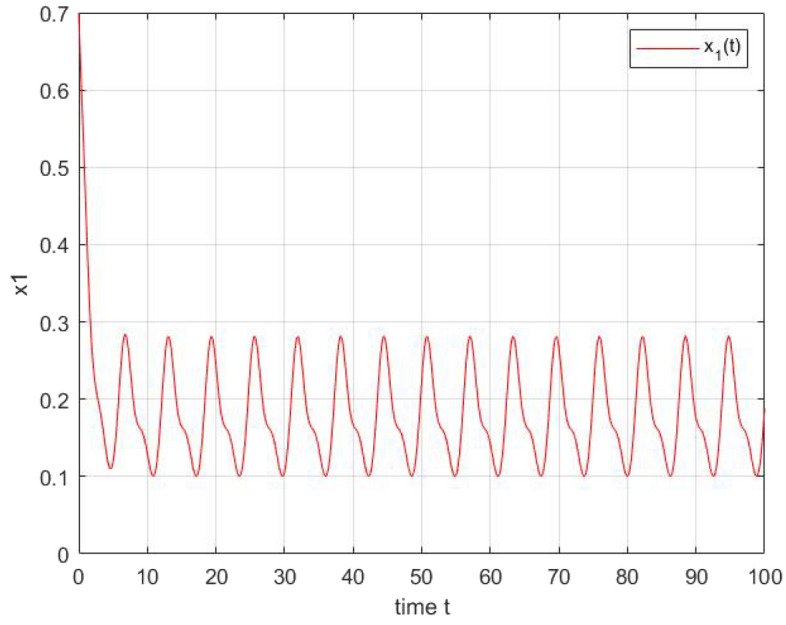
The state trajectories of $x_{1}(t)$.

**Figure 3:**
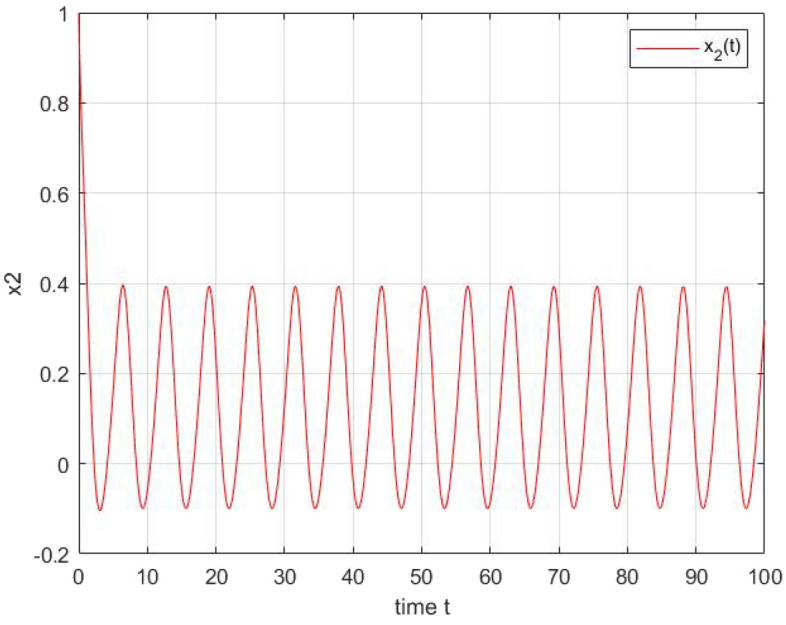
The state trajectories of $x_{2}(t)$.

We easily calculate

$$I-S=\left( \begin{array}{cc} 0.49 &{} 0.84\\ 0.65 &{} 0.07 \end{array}\right)$$. Thus, the conditions required in Theorem 1 are satisfied. When I (t) is a periodic function, in the view of Theorem 1, this neural network has at least one periodic solution. It is clear that $I-S$ is an M-matrix. Then theorem 4 holds and the system has a unique equilibrium point $x^{*}$, which is globally exponentially stable.

After simulation of these two systems using matlab Toobox,we obtain the graphical illustration Figs. 2 and  3 shows the periodic dynamic behaviors of the output of the two neurons which are in accordance with theoretical results.

To prove the effectiveness of our result on finite-time synchronization we consider the master system the above simulated example and the following system is the slave.

Let consider the following response RNN:

$$\begin{array}{c} \dot{y}_{i}(t)=-a_{i}x_{i}(t)+\overset{2}{\underset{j=1}{\sum }}b_{ij}f_{j}(y_{j}(t))+\overset{2}{\underset{j=1}{\sum }}c_{ij}g_{j}(y_{j}(t-\tau _{j})) +\overset{2}{\underset{j=1}{\sum }}p_{ij}(t)\overset{t}{\underset{-\infty }{\int }}k_{ij}(t-s)h_{j}(y_{j}(s))ds+u_{i}(t). \end{array}$$

We choose n=2 neurons and $u_{i}(t)=exp(-0.5 \times t)$ and the initial states $x=[0.5;0.2];y=[0.7;0.3];$

$$\begin{aligned} e_{i}(t) = x_{i}(t)- y_{i}(t), i = 1, 2. \end{aligned}$$

**Figure 4:**
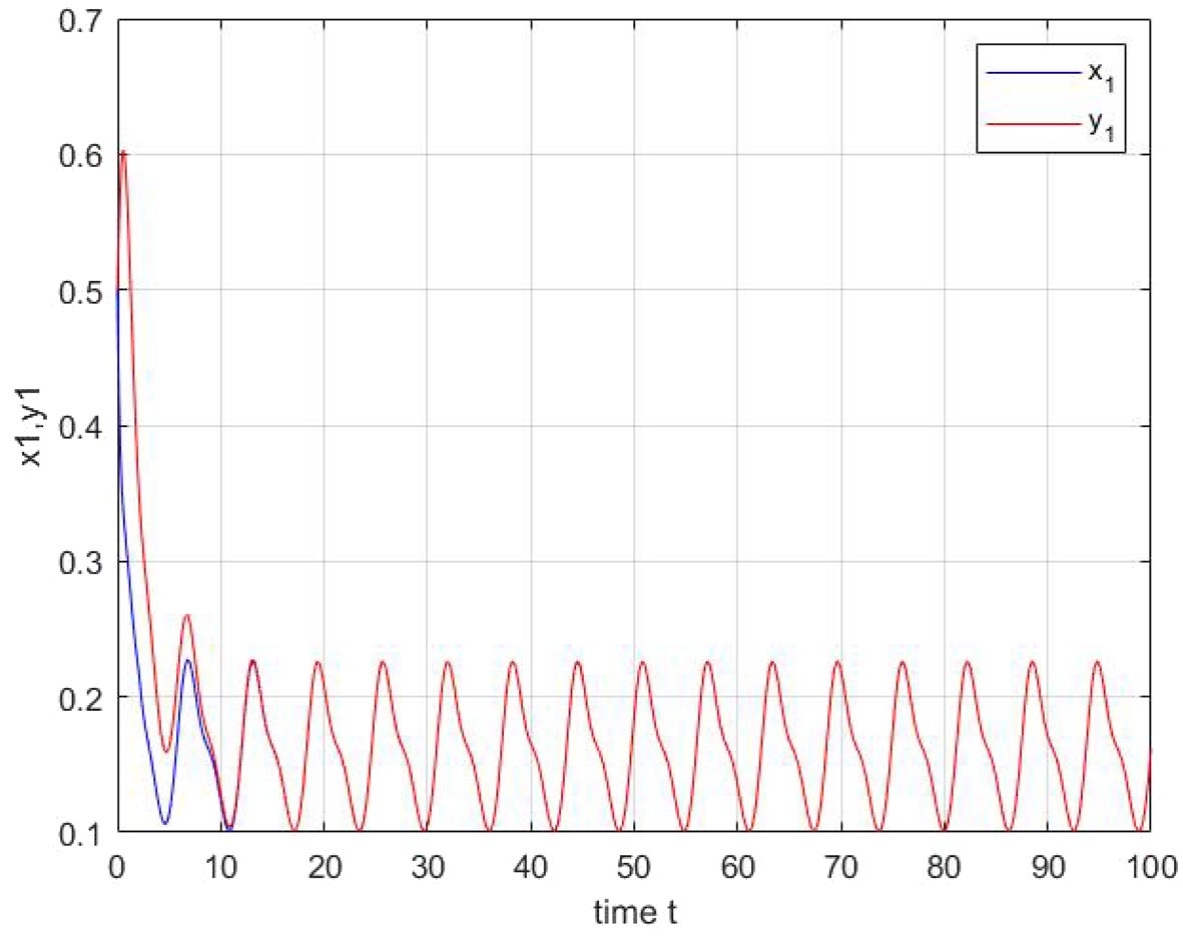
Time-domain behavior of the state variables x1(t) and y1(t).

**Figure 5:**
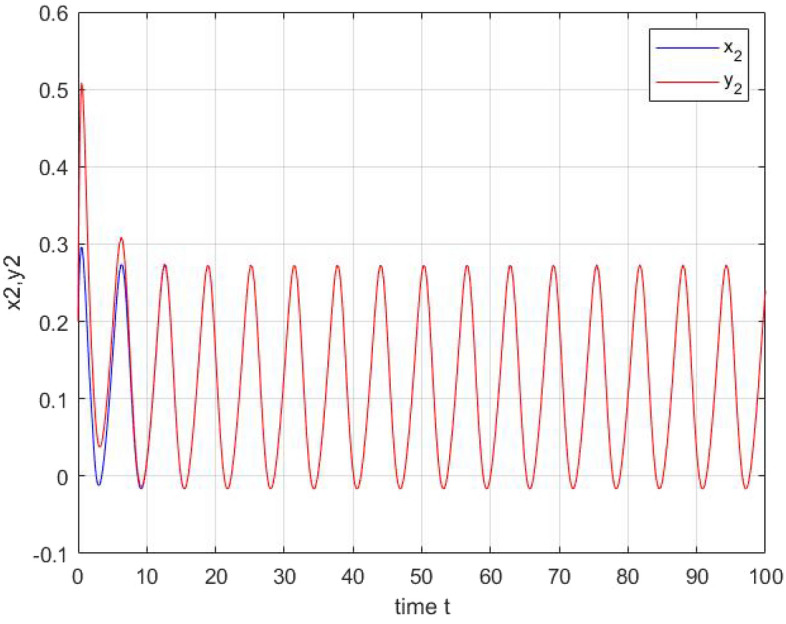
Time-domain behavior of the state variables x2(t) and y2(t).

**Figure 6:**
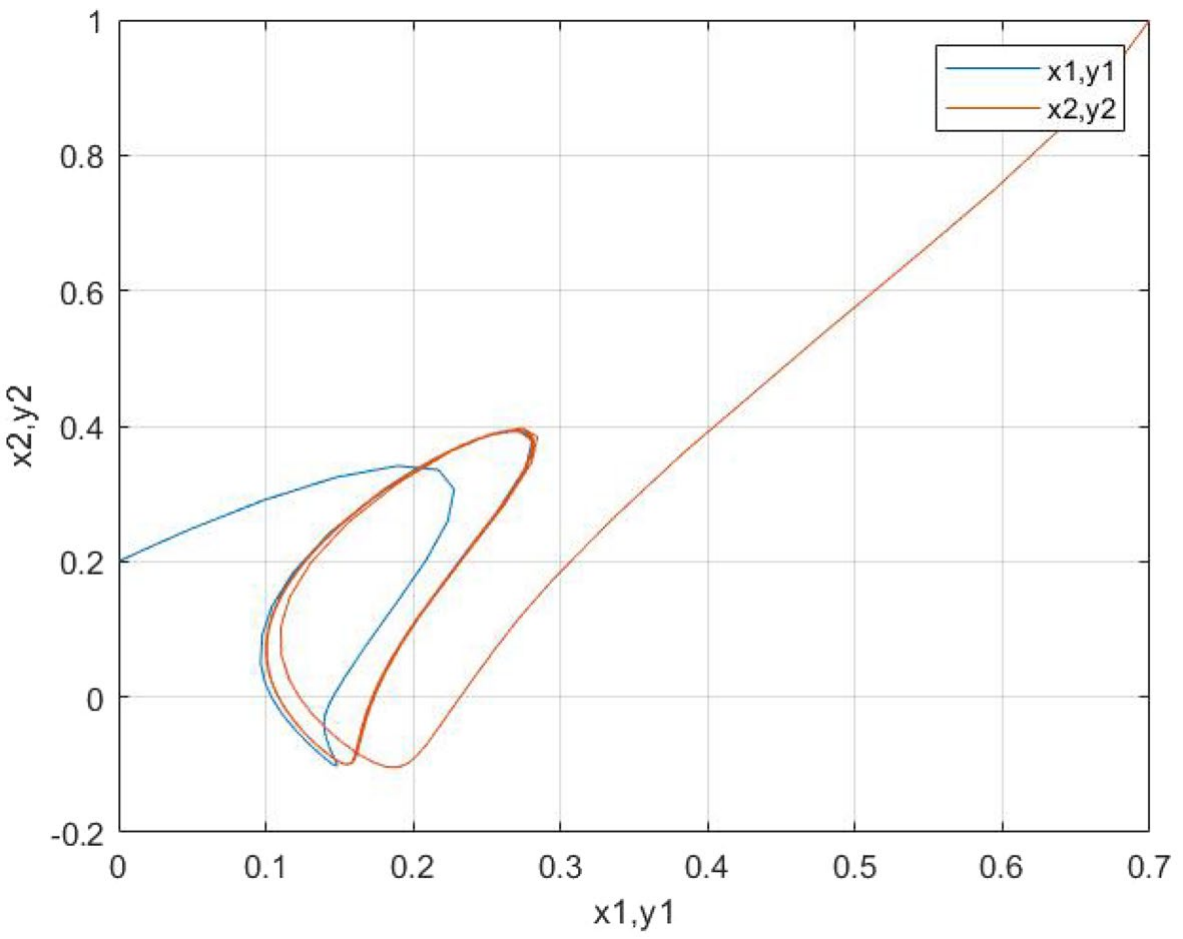
Phase plane behavior of the master system and the slave system.

**Figure 7:**
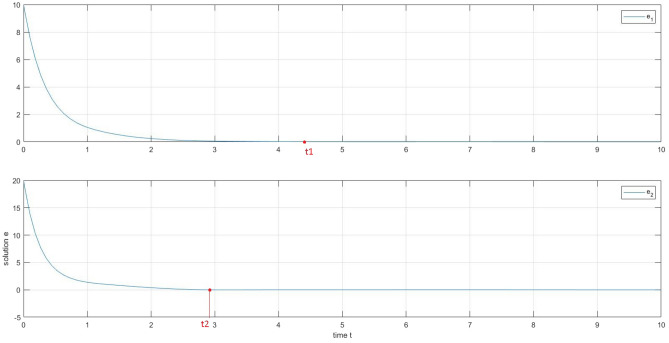
Finite-Time synchronization error.

We obtain in the following the simulation results: the two neurons tend to have the same trajectories in Figs. 4 and 5. Figs. 6 and 7 describes the time responses of finite-time synchronization errors and the trajectory turns to zero quickly as time goes and $t_{1}= 4.4$ and $t_{2}=2.9$.

## Conclusions

In this paper, we study a memristive recurrent neural networs by giving assumptions for the existence and uniqueness of periodic solution. In addition, we detemine sufficient conditions that ensure the global exponential stability of this solution. Further more, we garantee the finite-synchronization problem of delayed memristive by determining several assymptions.

Meanwhile, the theoretical proposed model can be tested in practical issues like brain computing interface, image processing, pattern recognition and intelligent control. In our ongoing future works, the proposed neural network model will be adjusted to analyze the electroencephalography (EEG) data for implementing continuous vigilance estimation using EEG signals acquired by wearable dry electrodes in both simulated and real driving environments. Also, MNN synchronization and EEG signals can be combined to study the brain dynamics at rest following a perturbation.

## Data Availability

The data that support the findings of this study are available from author Hajer Brahmi but restrictions apply to the availability of these data, which were used under license for the current study, and so are not publicly available. Data are however available from the authors upon reasonable request and with permission of the author Hajer Brahmi.
